# Identification of pathways that regulate circadian rhythms using a larval zebrafish small molecule screen

**DOI:** 10.1038/s41598-019-48914-7

**Published:** 2019-08-27

**Authors:** Eric A. Mosser, Cindy N. Chiu, T. Katherine Tamai, Tsuyoshi Hirota, Suna Li, May Hui, Amy Wang, Chanpreet Singh, Andrew Giovanni, Steve A. Kay, David A. Prober

**Affiliations:** 1 Division of Biology and Biological Engineering, California Institute of Technology, Pasadena, CA 91125 USA; 2 Centre for Cell and Molecular Dynamics, Department of Cell and Developmental Biology, University College London, London, WC1E 6BT United Kingdom; 3 PRESTO, JST, Nagoya, 464-8601 Japan; 4 Institute of Transformative Bio-Molecules, Nagoya University, Nagoya, 464-8601 Japan; 5 Sunovion Pharmaceuticals, Marlborough, MA 01752 USA; 6 Department of Neurology, Keck School of Medicine, University of Southern California, Los Angeles, CA 90089 USA; 7 Present Address: Program in Biological Sciences, Northwestern University, Evanston, IL 60201 USA; 8 Present Address: Department of Neurobiology, Northwestern University, Evanston, IL 60201 USA

**Keywords:** Screening, Circadian regulation

## Abstract

The circadian clock ensures that behavioral and physiological processes occur at appropriate times during the 24-hour day/night cycle, and is regulated at both the cellular and organismal levels. To identify pathways acting on intact animals, we performed a small molecule screen using a luminescent reporter of molecular circadian rhythms in zebrafish larvae. We identified both known and novel pathways that affect circadian period, amplitude and phase. Several drugs identified in the screen did not affect circadian rhythms in cultured cells derived from luminescent reporter embryos or in established zebrafish and mammalian cell lines, suggesting they act via mechanisms absent in cell culture. Strikingly, using drugs that promote or inhibit inflammation, as well as a mutant that lacks microglia, we found that inflammatory state affects circadian amplitude. These results demonstrate a benefit of performing drug screens using intact animals and provide novel targets for treating circadian rhythm disorders.

## Introduction

Circadian rhythms help ensure that physiological processes and behaviors occur at appropriate times during the 24-hour day/night cycle. These rhythms are generated and sustained at the cellular level by a transcriptional-translational negative-feedback loop that cycles with a period of approximately 24 hours, and are entrained by environmental cues such as light, food availability and temperature^1^. Molecular circadian oscillations in cells in different tissues and brain regions cycle with distinct phases, suggesting that non-cell autonomous mechanisms transmit circadian information throughout an animal^1^. While the suprachiasmatic nucleus (SCN) acts as a central circadian pacemaker to regulate circadian rhythms in mammals, it is unclear how the SCN transmits circadian information and if additional non-cell autonomous mechanisms exist. It is also unclear whether some aspects of the vertebrate circadian clock, which has primarily been studied using nocturnal rodents, differ from those in diurnal vertebrates such as humans. Thus, despite extensive research, mechanisms that regulate circadian rhythms remain incompletely understood.

Attempts to identify these mechanisms have primarily used two approaches. Genetic screens in model organisms have identified many components of the molecular circadian clock^1^. However, this approach may fail to detect genes that regulate aspects of the clock that do not affect the phenotype studied, or genes that have functionally redundant paralogs. Genetic screens are also difficult to perform using vertebrate animals. More recently, luminescent reporters have been used in cell culture to screen for cell autonomous factors that regulate the mammalian molecular circadian oscillator^2–8^. However, this approach lacks *in vivo* relevance and will not detect mechanisms that act non-cell autonomously or do not operate in the cell types used. Thus, alternative approaches could reveal novel mechanisms that regulate the circadian clock.

Most small molecule screens use *in vitro* or cell culture assays to identify drugs that bind a specific target or affect a specific process. However, these screens do not recreate the complex environment of whole animals and likely fail to identify some mechanisms that regulate the process under study. To overcome these limitations, we and others have used intact zebrafish as a vertebrate model system for small molecule screens^9^. This approach combines the *in vivo* relevance of whole-animal assays with moderate-throughput, low-cost drug screening. It also exploits several features of zebrafish larvae, including a relatively simple yet conserved vertebrate brain that lacks a mature blood-brain-barrier^10^, a small size that allows for screening in multi-well plates, and optical transparency that facilitates the use of luminescent reporters. Importantly, for the purposes of circadian research, the zebrafish molecular circadian oscillator closely resembles that of mammals^11^.

Here we describe a screen for small molecules that affect molecular circadian rhythms using a luminescent reporter in zebrafish larvae. We also monitor behavioral circadian rhythms using an assay that we previously used to identify drugs that regulate larval zebrafish locomotor behaviors^12^. We show that small molecules targeting pathways known to affect the circadian clock induce the expected circadian phenotypes in intact zebrafish. We also identify drugs that implicate novel pathways in regulating circadian rhythms *in vivo* that are absent in cultured cells. Finally, we show that inflammatory state affects circadian amplitude using both drugs and *xpr1b* mutant zebrafish, which lack microglia. These results reveal an unexpected role for the immune system in regulating the circadian clock.

## Results

### A screen for small molecules that affect molecular circadian rhythms in zebrafish larvae

**Figure 1:**
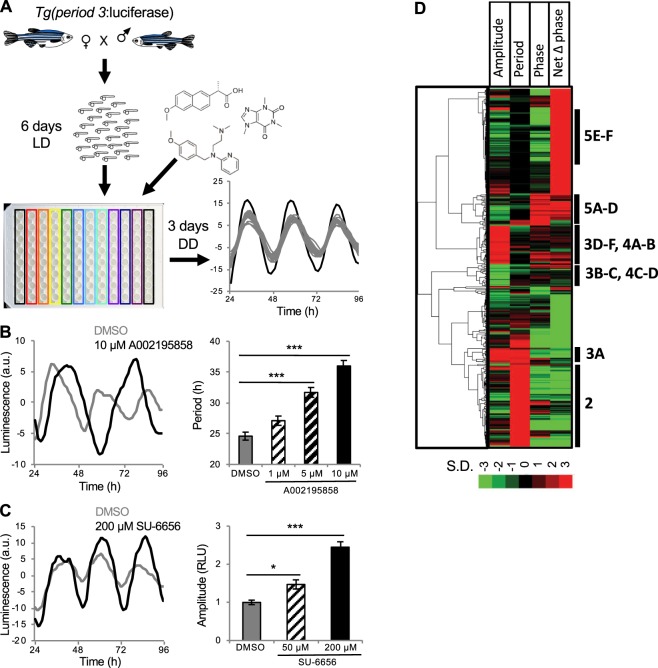
A screen for drugs that affect molecular circadian rhythms in *Tg(per3:luc)* zebrafish larvae. (**A**) Progeny from a homozygous *Tg(per3:luc)* to WT mating were raised for 6 days at 22 °C in 14:10 hour LD. Individual larvae were then added to each well of a 96-well plate, drugs or DMSO vehicle control was added to the water, and luminescence was monitored for 72 hours in DD. An example of a drug that increased amplitude (black line) compared to DMSO controls from multiple plates (gray lines) is shown. (**B**) A002195858, a CKI inhibitor that increases period length in mammalian cells, dose-dependently increased period length in zebrafish larvae compared to DMSO control. (**C**) SU-6656, a Src kinase inhibitor, dose-dependently increased amplitude in zebrafish larvae compared to DMSO control. Black and gray lines represent drug and DMSO control, respectively. Line graphs show mean and bar graphs show mean ± SEM for 8 animals. (**D**) Drugs that altered circadian amplitude, period length or phase by 3 or more standard deviations from the mean of same-plate DMSO controls, organized using hierarchical clustering. Bars and annotations on the right indicate figures with examples of phenotypes. See Methods for description of Net Δ Phase. Individual drugs in the clustergram can be viewed in Fig. S1. a.u. = arbitrary units. RLU = relative light units. *P < 0.05, ***P < 0.001 by ANOVA with Bonferroni correction.

A previous study described transgenic zebrafish in which the promoter for the *period3* gene regulates expression of firefly luciferase (*Tg(per3:luc)*), and showed that this line accurately reports molecular circadian rhythms in zebrafish larvae^13^. To test whether this line could be used to screen for small molecules that affect molecular circadian rhythms, we asked whether compounds that affect the circadian clock in cell culture induce similar effects in zebrafish larvae. We entrained *Tg(per3:luc)* larvae in 14:10 hour light:dark (LD) conditions for 6 days at 22 °C^13^. We then placed individual larvae into each well of a 96-well plate, added small molecules or DMSO vehicle control to each well, and monitored luminescence for 72 hours in constant darkness (DD) (Fig. 1A). To validate our assay, we first tested a drug that targets a pathway known to affect circadian period length. Pharmacological inhibition of casein kinase 1 (CK1) increases period length in mammalian cell culture^3,5,14^, rodents^5,15^ and zebrafish^5,15,16^, similar to some *ck1* mutant animals^17–20^. We tested a compound, A002195858, that inhibits CK1 *in vitro* (IC_50_ = 23 nM) and dose-dependently increases period length in mammalian cells (Fig. S2F), and found that it also dose-dependently increases period length in our larval zebrafish assay (Fig. 1B). We also found that the Src kinase inhibitor SU-6656^21^ dose-dependently increases circadian amplitude in our assay (Fig. 1C). These results indicate that *Tg(per3:luc)* larvae can be used to report drug-induced changes in molecular circadian rhythms, and that phenotypes observed in mammalian cells can also be observed in zebrafish larvae.

Encouraged by these results, we screened 6213 bioactive compounds, representing 3968 unique structures, from commercially available libraries. Eight larval zebrafish, raised as described above, were exposed to each compound at a concentration that ranged from 10 μM to 90 μM, depending on the library (most drugs were used at 30 μM, see Methods), for 72 hours in DD. To analyze luminescence data, we initially fit the data from individual larvae to a damped cosine curve using nonlinear least squares, as described for cell culture screens^2^. This approach worked well for strong phenotypes, but failed to capture subtle phenotypes and resulted in a high rate of false positives (as determined by manual examination of raw data), likely due to noise resulting from animal movement. To overcome these problems, we employed a wavelet-based time series analysis, which allows the removal of oscillations present at frequencies that are irrelevant to circadian rhythms, thus reducing noise^22^. We used a discrete wavelet transform to de-noise and de-trend the raw luminescence data, followed by a continuous wavelet transform to extract circadian parameters. This analysis improved identification of subtle phenotypes and reduced the number of false positives. We found that 354 compounds altered circadian period length, phase or amplitude by at least 3 standard deviations from the mean of same-plate DMSO controls. Using the values for amplitude, period length and phase, we generated a phenotypic fingerprint for each compound and used hierarchical clustering to organize compounds according to their fingerprint^12,23^ (Figs. 1D and S1). This analysis organized the dataset into clusters representing specific phenotypes. Drugs that induced phenotypes, as well as drugs known to affect the same pathways but not included in the screen, were obtained from an independent source and tested to validate the screen results, as described below.

### Small molecules that affect circadian period length via known mechanisms

**Figure 2:**
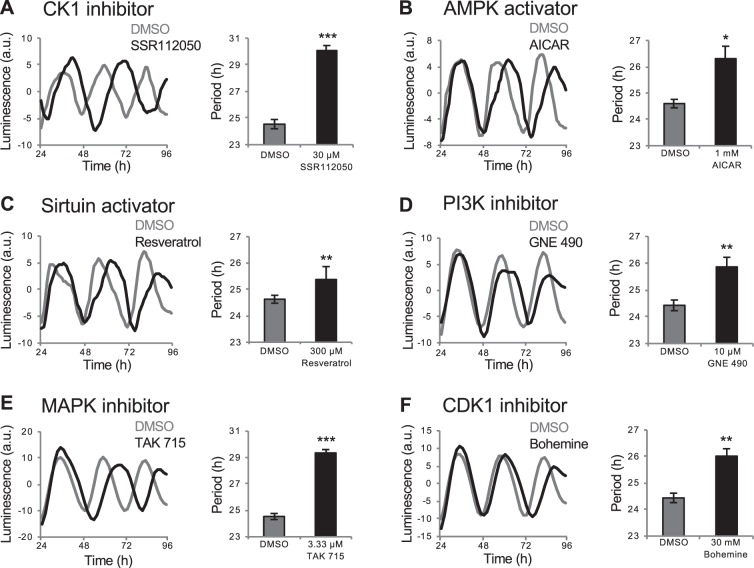
Drugs that increase *Tg(per3:luc)* luminescence circadian period length. Compounds that increased period length include CK1 inhibitor SSR112050 (**A**), AMPK activator AICAR (**B**), sirtuin activator resveratrol (**C**), PI3K inhibitor GNE 490 (**D**), P38 MAPK inhibitor TAK 715 (**E**) and CDK1 inhibitor bohemine (**F**). Black and gray represent drug and DMSO control, respectively. *P < 0.05, **P < 0.01, ***P < 0.001 by ANOVA with Tukey’s test.

We identified several categories of compounds that increased period length in zebrafish larvae **(**Fig. S2A), including drugs that increased period length in previous cell-based drug screens, and drugs that target proteins known to regulate period length in mammals. First, several studies have shown that CK1 regulates period length by phosphorylating period (Per) proteins, which affects their subcellular localization and promotes their degradation^24^. Accordingly, some mutations in *ck1* increase period length in *Drosophila* and mammals^17–20^, as do CK1 inhibitors in rodents^15^, cultured mammalian cells^3,5,14^ and zebrafish^5,16^. We found that the CK1 antagonist SSR112050 increased period length in our whole-larva assay (Figs. 2A and S2A). The screen included several additional drugs annotated as CK1 antagonists, but they were either toxic (i.e. caused morphological abnormalities or lethality) at the doses tested or resulted in very low amplitude rhythms, possibly due to a large period increase, which could not be fit using wavelet analysis, as previously reported in larval zebrafish^25^. We also identified several kinase inhibitors that increased period length but are not annotated as CK1 antagonists (Fig. S2A), but might affect the clock by inhibiting CK1 due to the promiscuous nature of these compounds for protein kinases^3^.

Second, activation of adenosine monophosphate-activated protein kinase (AMPK) leads to destabilization of cryptochrome 1 and an increase in period length in mammalian cell culture^26^. Conversely, AMPK mutation results in a shorter circadian period in mice^27^. Consistent with these results, we found that AICAR and metformin, which activate AMPK, increased period length (Figs. 2B and S2B). Studies in mammals have shown that SIRT1, which functions downstream of AMPK^28^, promotes the degradation of Per2^29^. Accordingly, inhibition of SIRT1 shortens the circadian period in mammalian cell culture^30^, and the SIRT1 activators resveratrol^31^ and isonicotinamide^32^ increased period length in our whole larva assay (Figs. 2C and S2C). Third, knockdown of phosphoinositide-3 kinase (PI3K) increases period length in a human cell line^4^, and we identified three PI3K inhibitors (GNE 490, LY-294002 and AS 252424) that increased period length in whole larval zebrafish (Figs. 2D, S2A and S2D). Fourth, inhibition of mitogen-activated protein kinases (MAPKs) increases period length in cultured chick pineal cells^33^ and mammalian cells^2^, and we identified a p38 MAPK inhibitor that increased period length in larval zebrafish (Fig. 2E). Fifth, inhibition of cyclin-dependent kinases (CDKs) increases period length in *Aplysia*^34^ and mammalian cell culture^2^, and we identified two CDK inhibitors (bohemine and roscovitine) that increased period length in larval zebrafish (Figs. 2F and S2E).

To confirm that drug-induced effects on period length were caused by changes to the molecular circadian oscillator, we used reverse transcription quantitative PCR (RT-qPCR) to measure *per1b* and *per3* levels in larvae raised and tested identically to those in the screen. We found that the CK1 inhibitor SSR112050 increased period length by an amount similar to that observed using the luminescence assay (Fig. S3A,B). We conclude that the luminescence assay provides an accurate measure of changes in period length.

### Small molecules that affect circadian amplitude via known and novel mechanisms

Many components of the circadian clock were identified using genetic screens that primarily assayed period length and phase, presumably because these clock parameters can be easily observed using developmental and behavioral assays^35^. Similarly, small molecule screens using cell culture have mainly identified drugs that affect period length and phase, although some drugs that affect amplitude have also been identified^7,36^. As a result, less is known about pathways that affect the amplitude of circadian oscillations. We were surprised to identify several categories of drugs that affect circadian amplitude in zebrafish larvae (Figs. S4A and S5A).

**Figure 3:**
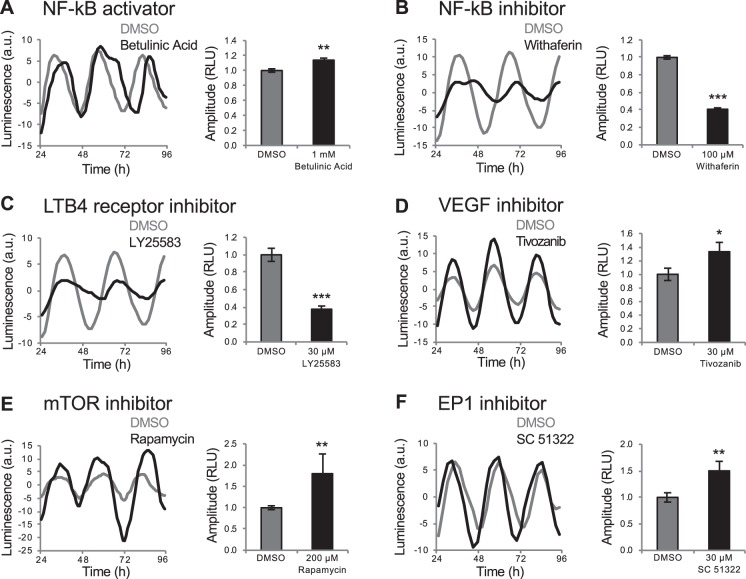
Drugs that affect *Tg(per3:luc)* luminescence circadian amplitude. NF-κB activator betulinic acid increased circadian amplitude (**A**). NF-κB inhibitor withaferin (**B**) and LTB4 receptor inhibitor LY25583 (**C**) decreased circadian amplitude. VEGF inhibitor Tivozanib (**D**), mTOR inhibitor rapamycin (**E**) and EP1 inhibitor SC 51322 (**F**) increased circadian amplitude. *P < 0.05, **P < 0.01, ***P < 0.001 by ANOVA with Tukey’s test.

First, we found that betulinic acid, which activates NF-κB signaling, caused a small but significant increase in circadian amplitude in zebrafish larvae (Fig. 3A). To determine whether NF-κB signaling is required to maintain normal amplitude levels, we tested the NF-κB inhibitor withaferin, and found that it caused a decrease in amplitude (Fig. 3B). We also identified leukotriene receptor inhibitors (LY25583 and pranlukast) that decreased amplitude (Figs. 3C and S4B), consistent with the observation that leukotriene B4 activates NF-κB signaling^37^. These results suggest that NF-κB signaling is necessary for normal circadian amplitude levels. Consistent with our findings, mammalian cells lacking the NF-κB subunit RelB exhibit smaller amplitude oscillations of *per1*, *per2*, and *per3*^38^.

Second, we found that a vascular endothelial growth factor receptor (VEGFR) inhibitor (Tivozanib/AV951) increased circadian amplitude (Fig. 3D). While *vegf* expression shows a circadian oscillation in hypoxic tumor cells^39^ and clock genes affect VEGF-dependent angiogenesis in zebrafish^40^, VEGF signaling has not been implicated in regulating the circadian clock. Third, we identified one mammalian target of rapamycin (mTOR) inhibitor and two prostaglandin receptor antagonists (SC 51322 and SC 19220) that increased amplitude (Figs. 3E,F and S4A). These pathways have not been implicated in regulating circadian amplitude, but inhibiting them can block light-induced circadian phase shifts in rodents^41,42^. These results suggest that an increase in amplitude may decrease susceptibility to phase resetting.

### Inflammation regulates circadian amplitude

**Figure 4:**
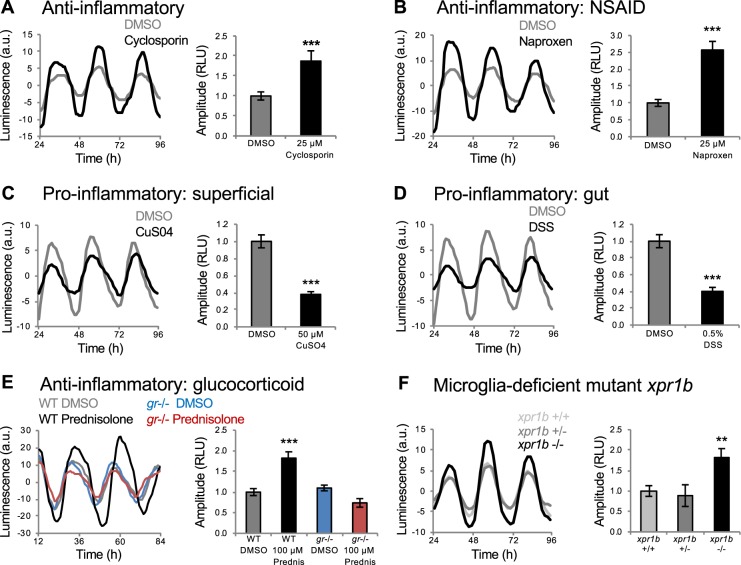
Inflammation regulates *Tg(per3:luc)* luminescence circadian amplitude. Anti-inflammatory compounds, including cyclosporin (**A**) and the NSAID naproxen (**B**) increased circadian amplitude. Pro-inflammatory compounds, including CuSO4 (**C**), which causes superficial inflammation, and DSS (**D**), which causes inflammation of the gut, decreased circadian amplitude. (**E**) Increased amplitude induced by the glucocorticoid prednisolone was abolished in *gr* mutant larvae. (**F**) *xpr1b*−/− larvae (n = 47) exhibited increased circadian amplitude compared to their *xpr1b*+/− (n = 93) and *xpr1b*+/+ (n = 52) siblings. Data are from 4 independent *xpr1b* experiments combined. **P < 0.01, ***P < 0.001 by ANOVA with Tukey’s test.

We identified a cluster of functionally diverse anti-inflammatory compounds that increased circadian amplitude (Figs. 4 and S5A). These include cyclosporin (Fig. 4A), non-steroidal anti-inflammatory drugs (NSAIDs) (Figs. 4B and S5A), steroidal glucocorticoids (Figs. 4E and S5A), phosphodiesterase (PDE) inhibitors (Fig. S5A,B) and other drugs that inhibit inflammation, including an mTOR inhibitor (Fig. 3E), a Src family kinase inhibitor (Fig. 1C) and a retinoid (Fig. S5D)^43–45^. We confirmed this phenotype for the NSAID naproxen using RT-qPCR (Fig. S3C,D). This cluster also contained a focal adhesion kinase (FAK) inhibitor (Fig. S5E), consistent with the observation that *fak* knock-out mice have reduced inflammation in a wound-healing model^46^. Based on these results, we hypothesized that induction of inflammation would cause a decrease in circadian amplitude. We tested this hypothesis by treating *Tg(per3:luc)* larvae with compounds known to induce inflammation in zebrafish larvae, including CuSO_4_, which induces inflammation in superficial tissues^47^, and dextran sodium sulfate (DSS) and trinitrobenzene sulfonic acid (TNBS), which induce inflammation of the digestive tract^48^. All three compounds decreased circadian amplitude in our assay (Figs. 4C,D and S5C), suggesting that the inflammatory system normally regulates the amplitude of circadian oscillations. While the circadian clock is known to regulate the immune system^49^, to our knowledge this is the first evidence that inflammatory state regulates the circadian clock.

Steroidal glucocorticoids inhibit inflammation via the glucocorticoid receptor (GR). To ask whether these drugs increase circadian amplitude via the GR, we tested them on *gr* mutant larvae^50^. While *gr* mutant larvae exhibited normal circadian rhythms (Fig. 4E), the increased circadian amplitude induced by the glucocorticoid prednisolone was abolished in *gr* mutants (Fig. 4E), suggesting that prednisolone increases circadian amplitude by signaling via the GR.

As an alternative approach to test whether the inflammatory system regulates circadian amplitude, we monitored *per3:luc* luminescence in *xpr1b* mutant zebrafish^51^. These animals lack microglia, brain-resident macrophages that are the main source of inflammation in the brain^52^. We found that *xpr1b*−/− animals exhibit increased circadian amplitude compared to their *xpr1b*+/− and *xpr1b*+/+ siblings (Fig. 4F). This result is consistent with our pharmacology data and suggests that microglia contribute to the regulation of circadian amplitude.

### Small molecules that affect circadian phase via known and novel mechanisms

**Figure 5:**
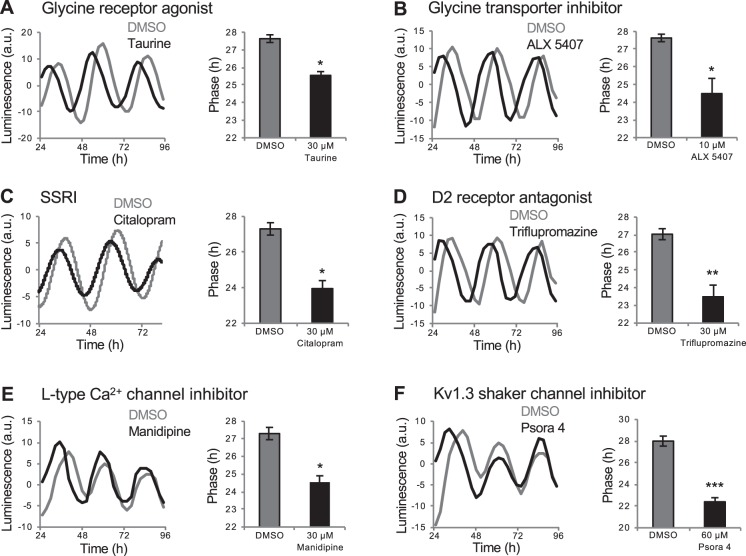
Drugs that affect *Tg(per3:luc)* luminescence circadian phase. Glycine receptor agonist taurine (**A**) and glycine transporter inhibitor ALX 5407 (**B**) caused phase advances. SSRI citalopram (**C**) and D2 receptor antagonist triflupromazine (**D**) induced phase advances. L-type calcium channel inhibitor manidipine (**E**) and Kv1.3 shaker channel inhibitor psora 4 (**F**) induced phase advances. Psora 4 only affected phase during the first circadian oscillation (**F**). *P < 0.05, **P < 0.01, ***P < 0.001 by ANOVA with Tukey’s test.

We identified several categories of drugs that altered circadian phase, several of which are associated with the nervous system and were not identified in previous cell culture screens. However, some of these drugs affect pathways shown to affect the circadian system using molecular or behavioral assays. First, we identified a glycine receptor agonist (Fig. 5A) and a glycine transporter inhibitor (Fig. 5B), both of which result in stimulation of glycine receptor signaling, that caused a phase advance. Consistent with this observation, glycine receptor agonists advance the phase of neuronal activity in the mouse SCN when applied during the subjective day^53^. Second, we identified several selective serotonin reuptake inhibitors (SSRIs), which increase serotonin signaling, that caused a phase advance (Figs. 5C and S6). Similarly, serotonin receptor antagonists can block behaviorally-induced phase advances in hamsters^54^. Third, we identified several dopamine D2 receptor antagonists (triflupromazine, butaclamol, clozapine) that caused a phase advance (Figs. 5D and S6). D2 receptor antagonist treatment can decrease clock gene expression in the mouse brain^55^, but effects on circadian phase have not been shown.

We also identified phase-shifting drugs that affect ion channels. For example, we identified several L-type calcium channel inhibitors that advanced circadian phase (Figs. 5E and S6). These channels are highly expressed in the mammalian SCN^56^ but have not been shown to affect circadian rhythms. We also identified a Kv1.3 shaker channel inhibitor that caused a phase advance (Fig. 5F). Kv1.3 shaker channels have been implicated in regulating sleep in *Drosophila*^57^, rodents^58^ and zebrafish^12^, but have not been shown to affect the circadian clock. The effects of L-type and Kv1.3 channel antagonists are likely specific to each receptor type because these drugs induce distinct behavioral phenotypes in zebrafish larvae^12^. The Kv1.3 shaker channel inhibitor only caused a phase shift during the first circadian oscillation, suggesting that it may affect the circadian clock via a mechanism distinct from that of other phase shifting compounds.

### Effects of drugs that increase molecular circadian period length and amplitude on behavioral circadian rhythms

**Figure 6:**
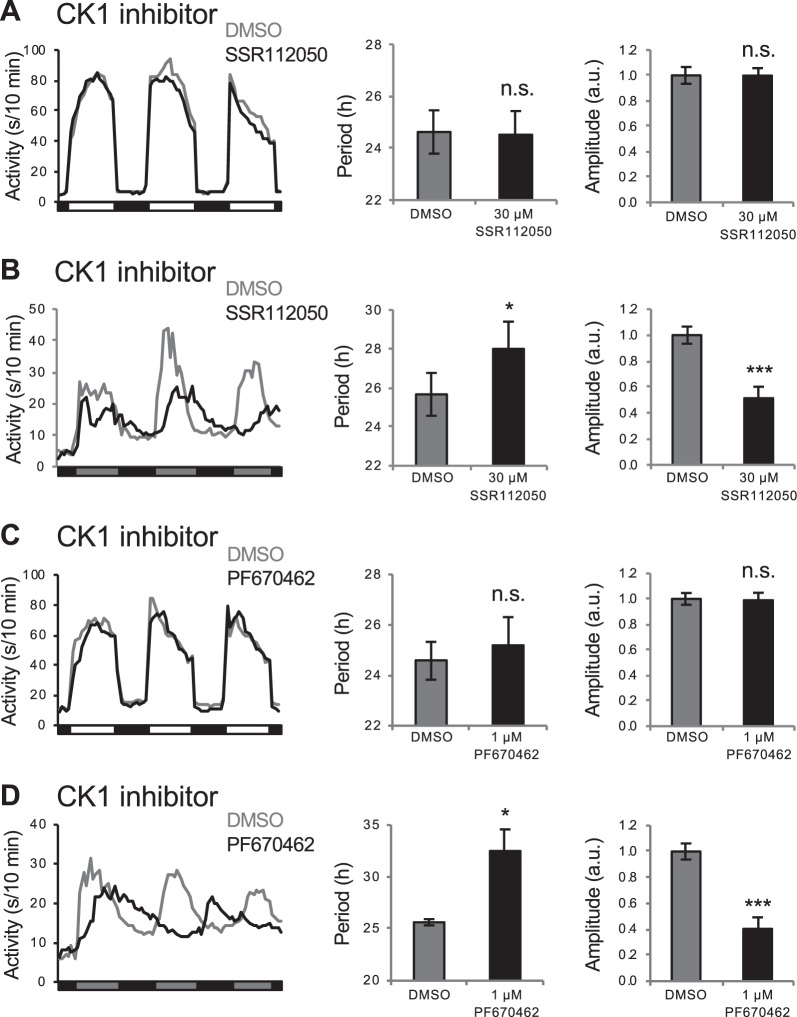
Drugs that elongate the *Tg(per3:luc)* luminescence circadian period also elongate the locomotor activity circadian period in free-running conditions. In LD, CK1 inhibitors SSR112050 and PF670462 do not affect locomotor activity period length or amplitude (**A**,**C**). In contrast, both drugs increase locomotor activity period length and decrease locomotor activity amplitude in entrained animals that were shifted to DD (**B**,**D**). Number of animals: (**A**) DMSO n = 42, SSR112050 n = 34. (**B**) DMSO n = 35, SSR112050 n = 31. (**C**) DMSO n = 32, PF670462 n = 32. (**D**) DMSO n = 34, PF670462 n = 30. n.s. = not significant, *P < 0.05, ***P < 0.001 by ANOVA with Tukey’s test.

Zebrafish larvae that are entrained in LD and shifted to DD maintain circadian rhythms of locomotor activity^13,59,60^. To test whether compounds that affect molecular circadian rhythms have similar effects on behavioral rhythms, we raised and entrained larvae in the same manner as for the luminescence assay, and then monitored locomotor activity in LD or DD^61^. Measurement of behavioral circadian period length in LD is confounded by the direct effects of light and dark on behavior, known as masking^62^, and two CK1 antagonists had no apparent effect on locomotor activity period length in LD (Fig. 6A,C), as expected. These drugs also had no effect on behavioral circadian amplitude in LD (Fig. 6A,C). However, similar to their effects on molecular circadian period, the two CK1 inhibitors elongated the locomotor activity circadian period in DD **(**Fig. 6B,D). These compounds also decreased locomotor activity during the subjective day in DD, thus decreasing the circadian locomotor activity amplitude (Fig. 6B,D), as previously shown for other CK1 inhibitors^25^.

**Figure 7:**
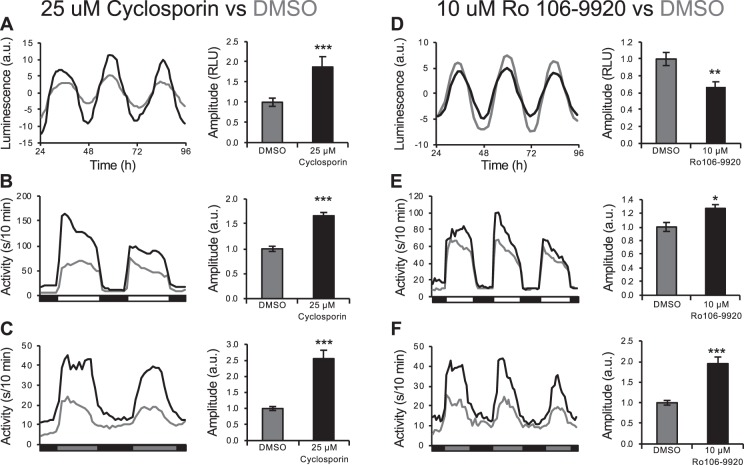
Drugs that affect *Tg(per3:luc)* luminescence circadian amplitude do not necessarily similarly affect locomotor activity circadian amplitude. Anti-inflammatory drug cyclosporin (**A**) and NF-κB inhibitor Ro 106-9920 (**D**) increase and decrease larval *per3:luc* luminescence amplitude, respectively. However, both compounds increase locomotor activity circadian amplitude in LD (**B,E**) and in entrained animals shifted to DD (**C,F**). Number of animals: (**B**) DMSO n = 36, cyclosporin n = 39. (**C**) DMSO n = 45, cyclosporin n = 34. (**E**) DMSO n = 45, Ro 106-9920 n = 37. (**F**) DMSO n = 45, Ro 106-9920 n = 40. *P < 0.05, **P < 0.01, ***P < 0.001 by ANOVA with Tukey’s test. Luminescence data shown in (**A**) is the same as that shown in Fig. 4A.

We also tested the effects of the anti-inflammatory cyclosporin and the NF-κB inhibitor Ro 106-9920, which increased and decreased molecular circadian amplitude in the luminescence assay, respectively. While these drugs had opposite effects on the amplitude of molecular rhythms (Fig. 7A,D), they both increased the circadian locomotor activity amplitude in LD and DD (Fig. 7B,C,E,F). These results suggest that manipulation of molecular circadian period length results in similar effects on behavioral period length, whereas effects on molecular circadian amplitude do not necessarily result in similar effects on behavioral amplitude.

### Identification of drugs that affect molecular circadian rhythms in zebrafish larvae but not in cultured zebrafish or mammalian cells

Several drugs that affected molecular circadian rhythms in larval zebrafish did not induce phenotypes in mammalian cell culture^2,3,5–7^. This discrepancy could be explained if these drugs affect circadian rhythms in a non-cell autonomous manner, requiring intact tissues or whole animals for their effects to be observed. Alternatively, these drugs might act via mechanisms absent in the mammalian cell types used, or may affect zebrafish but not mammals. Our observations might also be specific to the *per3* promoter, and not reflective of general effects on the molecular circadian oscillator. We used three strategies to distinguish between these possibilities.

**Figure 8:**
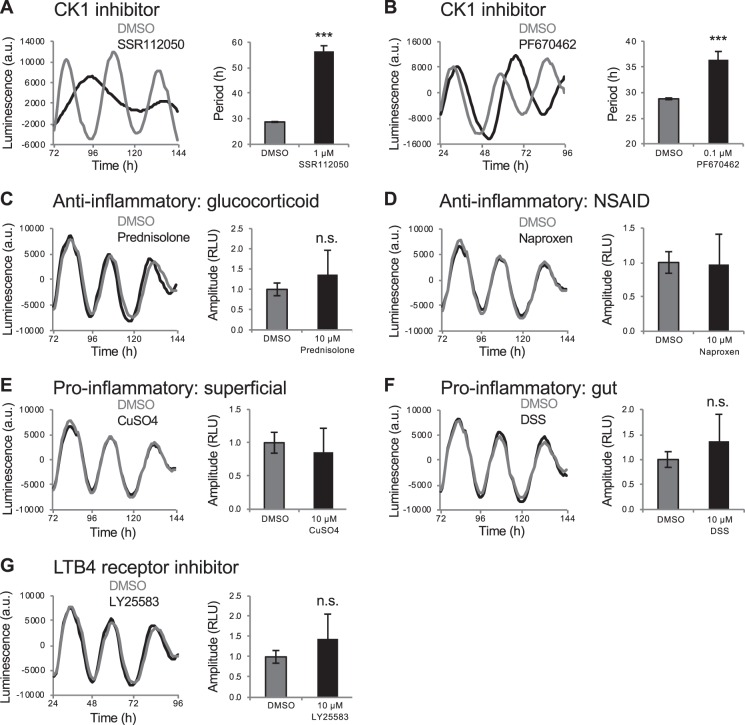
Compounds that affect amplitude in *Tg(per3:luc)* larvae but not in *per3:luc* cells. CK1 inhibitors SSR112050 (**A**) and PF670462 (**B**) both increased period length in *per3:luc* cells, similar to their effects on *Tg(per3:luc)* larvae. Drugs that increased (**C**,**D**) or decreased (**E**–**G**) amplitude in *Tg(per3:luc)* larvae had no effect in *per3:luc* cells. n.s. = not significant, ***P < 0.001 by ANOVA with Tukey’s test.

First, to directly compare phenotypes in intact animals to primary cells derived from those animals, we dissociated *Tg(per3:luc)* embryos at 24 hours post fertilization, added the dissociated cells to 96-well plates, entrained them for up to 3 days in LD, and then monitored luminescence in DD. We found that the CK1 antagonists SSR112050, PF670462 and A002195858 increased period length (Figs. 8A,B and S7A), consistent with findings in mammalian cell culture^4,5,7^. In contrast, some drugs that increased period length in zebrafish larvae, including AICAR (AMPK activator), resveratrol (SIRT1 activator) and LY-294002 (PI3K inhibitor), had no effect on period length in cultured *per3:luc* cells (Fig. S7A). Similarly, compounds that affected amplitude in larvae did not cause similar effects in cultured cells, although the amplitude data was noisier than that for period length (Figs. 8C–G and S7B). Some compounds decreased amplitude in cells (Fig. S7B), but in each case this was associated with drug-induced toxicity, and thus fewer cells and a smaller luminescent signal. Thus, many compounds that affected circadian oscillations in zebrafish larvae failed to induce similar phenotypes in primary cells acutely prepared from these larvae.

Second, we used a *period1b:luciferase* (*per1b:luc*) zebrafish cell line^63^, which exhibits circadian luciferase expression. Similar to *per3:luc* cells, CK1 antagonists increased period length in *per1b:luc* cells, whereas compounds that increased period length in *Tg(per3:luc)* larvae but not in *per3:luc* cells had no effect (Fig. S8A). Compounds that affected amplitude in larvae did not cause similar effects in *per1b:luc* cells (Fig. S8B). Similar to the *per3:luc* cell results, decreased amplitude was observed for some compounds due to drug-induced toxicity. These results agree with those obtained using acutely prepared *per3:luc* cells, suggesting that the *per3:luc* results are not specific to that promoter.

Third, we used a *Bmal1:dluciferase* (*Bmal1:dluc*) U2OS (human osteosarcoma) cell line that has been used in cell-based circadian rhythm screens^2–5,7^. CK1 antagonists that increased period length in zebrafish larvae and cultured zebrafish cells had similar effects in mammalian cells, whereas drugs that increased period length in *Tg(per3:luc)* larvae, but not in *per3:luc* cells, had no effect (Fig. S9A). Similar to the zebrafish cell lines, drugs that affected amplitude in larvae did not cause similar effects in *Bmal1:dluc* cells (Fig. S9B). Decreased amplitude was observed for some compounds, again due to drug-induced toxicity.

These results show that most drugs that affect amplitude in zebrafish larvae fail to do so in cultured zebrafish or mammalian cells. This observation is consistent with the hypothesis that these compounds act in a non-cell autonomous manner and/or require an intact animal to exert their effects on the circadian clock. In either case, the larval zebrafish screen identified novel pathways that regulate circadian rhythms, demonstrating a benefit of using intact animals for small molecule screens.

## Discussion

Using zebrafish larvae, we performed the first screen for small molecules that affect molecular circadian rhythms in intact animals. This approach identified signaling pathways and biological processes that affect molecular circadian oscillations that were not identified in previous screens that used developmental or behavioral phenotypes in model organisms. Surprisingly, we identified several classes of drugs that were not identified in previous cell culture-based drug screens, even though those screens tested many more drugs^5,7^, including the some of the drugs that we tested. For example, we found that drugs known to inhibit or promote inflammation in zebrafish increase or decrease the amplitude of molecular circadian oscillations, respectively. Consistent with these observations, *xpr1b* mutant zebrafish^51^, which lack microglia and thus lack the main form of active immune defense and inflammation in the brain^52^, exhibit increased circadian amplitude. While the circadian clock has been shown to affect immune system function^64^, our results suggest that the immune system, in turn, feeds back to regulate the circadian clock. Drugs that affected the amplitude of molecular circadian oscillations in zebrafish larvae failed to induce similar phenotypes in a mammalian cell line^2^. These compounds were also inactive in cells derived from *Tg(per3:luc)* embryos and in a zebrafish *per1b:luc* cell line, suggesting that the failure of these compounds to induce circadian phenotypes in mammalian cells is not due to differences between zebrafish and mammals, but rather is due to differences between intact animals and cultured cells. For example, inflammation may affect circadian rhythms in a non-cell autonomous manner, perhaps requiring immune cells or inflammatory processes. This possibility is supported by our observation that *xpr1b* mutant zebrafish, which lack microglia, exhibit an increased amplitude of molecular circadian oscillations. Our observation that anti-inflammatory compounds acting via diverse mechanisms induce similar effects on circadian oscillations suggests the effect is due to a basic and general feature of inflammation. Further studies are needed to understand how inflammatory state affects circadian amplitude at the cellular and molecular levels.

Surprisingly, we did not identify novel pathways that affect circadian period length, suggesting that this aspect of circadian clock regulation has already been extensively characterized. We also failed to identify compounds that decreased period length, suggesting that the circadian clock may be less susceptible to perturbations that shorten period length compared to those that increase it. However, previous drug- and RNAi-based screens in mammalian cells identified similar numbers of compounds and genes that increased and decreased period length^2,4,7^. A particularly interesting comparison is our screen with that of Hirota *et al*.^2^, which screened the LOPAC drug library using the same *Bmal1:dluc* U2OS human cell line as our study. While both screens identified the same period-lengthening compounds, Hirota *et al*. also identified compounds that shortened period length, but these drugs had no effect in zebrafish larvae. This discrepancy suggests that period shortening mechanisms affected by these drugs in human cells may not operate in zebrafish, may only occur in certain cell types, or may not have significant effects in the context of an intact animal.

In addition to identifying novel pathways that regulate circadian rhythms, our results provide a strong rationale for performing small molecule screens using intact animals, since it allows the identification of mechanisms that do not operate in dissociated cells or are not present in particular cell types. Thus, despite the relatively modest number of compounds that can be screened using zebrafish larvae compared to cell culture, this can be a fruitful approach to identify mechanisms that regulate a process of interest that cannot be identified using higher-throughput cell-based or *in vitro* screening approaches. However, a caveat to this approach is that some drugs may only affect certain cell types or tissues, or may induce different effects in different tissues, which cannot be resolved by whole-animal luminescence monitoring. Future studies using *in situ* hybridization with probes specific for circadian clock genes, or using animals with tissue-specific circadian luminescence reporters, are needed to explore potential tissue-specific effects.

Finally, we note that circadian amplitude decreases with age in many animals, resulting in disrupted circadian rhythms^65^. Humans with disrupted circadian rhythms are at increased risk for several neurological disorders, and circadian dysfunction plays a causal role in some cases of these disorders^66–68^. It has therefore been proposed that maintaining robust circadian rhythms during aging may delay or reduce the severity of some neurological disorders^65^. The amplitude-modulating drugs identified in our screen can be used to directly test this hypothesis.

## Methods

### Transgenic and mutant zebrafish

The *Tg(per3:luc)* transgenic line^13^, *gr* mutant^50^ and *xpr1b* mutant^51^ have been previously described. Zebrafish experiments and husbandry followed standard protocols^69^ in accordance with, and approved by, Caltech Institutional Animal Care and Use Committee guidelines.

### Small molecules

Small molecules from the following libraries were obtained from the Harvard Institute of Chemistry and Cell Biology and screened at the following concentrations: (1) Sigma LOPAC (30 μM, 1280 compounds), (2) Biomol 4 FDA Approved library (2 mg/mL, 10–90 μM, 640 compounds), (3) NINDS Custom Collection 2 (30 μM, 1040 compounds), (4) Biomol 3 – ICCB Known Bioactives (30 μM, 480 compounds), (5) NIH Clinical Collection 1 (30 μM, 446 compounds), (6) NIH Clinical Collection 2 (30 μM, 281 compounds), (7) EMD Kinase Inhibitor library (30 μM, 244 compounds), (8) MSDiscovery 1 Collection (30 μM, 270 compounds), (9) Microsource 1 US Drug Collection (30 μM, 1040 compounds), (10) SYNnthesis Kinase Inhibitor 1 library (30 μM, 288 compounds), (11) ChemBridge GPCR library (30 μM, 250 compounds). Drugs for retesting were obtained from Tocris, Sigma-Aldrich and Cayman Chemical, with each drug tested on 8 animals, unless otherwise indicated. In all figures, line graphs represent mean and bar graphs represent mean ± SEM.

### Validation of CK1 inhibitor A002195858 in mammalian cell culture

Rat-1 fibroblasts stably transfected with a *mPer1-luc* reporter construct were synchronized and then released into serum-free media containing 0.3% DMSO alone or the small molecule A002195858 (synthesized by Aventis Project Chemists) in 0.3% DMSO. Plates were sealed and immediately placed in a 37 °C incubator. Luminescence was measured at 30–40 minute intervals for 5–6 days using a TopCount scintillation counter (Packard). A002195858 was tested at four concentrations in triplicate.

### Zebrafish luminescence experiments

Larval zebrafish harboring a *per3:luc* reporter^13^ were raised on a 14:10 hour LD cycle at 22 °C with lights on at 9 a.m. and off at 11 p.m. At 6 days post fertilization, individual larvae were placed into each well of 96-well plates (T-2996–075, Greiner), containing 50 μL Holtfreter’s solution (59 mM NaCl, 0.67 mM KCl, 0.76 mM CaCl_2_, and 2.4 mM NaHCO_3_, pH 7.9), 0.5 mM D-luciferin (L8220, Biosynth Chemistry and Biology) and 0.013% Amquel Instant Water Detoxifier (Kordon). Small molecules were added by pipetting a 10 mM stock solution (in DMSO) into each well, and plates were sealed with an optical adhesive film (4311971, Applied Biosystems). Bioluminescence in each well was recorded for 3 seconds at intervals ranging from every hour to every 2.83 hours, depending on the number of plates being recorded, for 100 hours in DD using a plate reader (M1000 Pro, Tecan). We observed no difference in the sensitivity of the assay when data was sampled every hour compared to every 2.83 hours. A robotic plate stacker was used to assay up to 30 plates during each experiment. A drug and DMSO control were each tested on 8 animals in each experiment. This number of animals was a compromise between data robustness and screen throughput, and was based on initial experiments using drugs known to affect period or amplitude, in which we found that 8 animals was sufficient to reliably detect relatively small phenotypes. Potential hits from the screen were retested using drugs obtained from an independent source in at least 3 independent experiments.

### *per3*:luc acute cell culture assay

Embryos were raised to 24 hours post fertilization at 28 °C before being bleached, manually dechorionated, and then dispersed with trypsin-EDTA (Invitrogen) and trituration through a 1 mL pipette tip. Dispersed cells were then filtered through a cell strainer (Falcon, 40 um), plated at approximately one embryo per well into 96 well plates and cultured in Leibovitz’s L15 medium with 15% fetal calf serum, 100 IU/mL penicillin, 0.1 mg/mL streptomycin, and 0.2 mg/mL gentamycin. Primary cells were maintained for up to 3 days on a 14:10 hour LD cycle at 28 °C with lights on at 9 a.m. and off at 11 p.m., and then transferred into DD for at least 3 days. Cell media contained 0.5 mM luciferin (Promega) and small molecules for testing, and plates were sealed with an optical adhesive film (Topseal, PerkinElmer). Bioluminescence in each well was recorded for 10 seconds approximately every hour for the duration of the experiment using a scintillation counter (Packard TopCount NXT).

### Luminescence assays using established cell lines

Luminescence assays were performed as described for *per1b:luc*^70^ and *Bmal1:dluc* cell lines^2^.

### Behavioral experiments

Videotracker experiments were performed as described^61^. Larval zebrafish were raised on a 14:10 hour LD cycle at 22 °C with lights on at 9 a.m. and off at 11 p.m. On the sixth day of development, individual larvae were placed in each well of a 96-well plate (7710-1651, Whatman) containing 650 μL of E3 embryo medium (5 mM NaCl, 0.17 mM KCl, 0.33 mM CaCl_2_, 0.33 mM MgSO_4_, pH 7.4). Plates were sealed with an optical adhesive film (4311971, Applied Biosystems) to prevent evaporation. Locomotor activity was monitored using an automated videotracking system (Viewpoint Life Sciences) with a Dinion one-third inch Monochrome camera (Dragonfly 2, Point Grey) fitted with a variable-focus megapixel lens (M5018-MP, Computar) and infrared filter. The movement of each larva was recorded using the quantization mode. The 96-well plate and camera were housed inside a custom-modified Zebrabox (Viewpoint Life Sciences) that was continuously illuminated with infrared lights and with customizable white light. The 96-well plate was housed in a chamber filled with recirculating water to maintain a constant temperature of 28.5 °C. The parameters used for detection, which were empirically determined, were: detection threshold, 15; burst, 29; freeze, 3; bin size, 60 seconds. At the end of each experiment, each well was examined and those containing bubbles (introduced during the sealing process) or more or less than one larva were excluded from analysis. We tested at least 20 animals for each condition, as we previously found this to be sufficient to generate robust data. Data was processed using custom PERL and Matlab (The Mathworks, Inc) scripts. Statistical tests were performed using Matlab.

### Curve fitting and wavelet analysis

Curve fitting analysis was performed as described^2^. Briefly, raw luminescence time series data from individual wells were fit to a damped cosine curve using nonlinear least squares. Due to transient luminescence changes caused when larvae are first added to the well, the first ~15–20 hours of data was filtered out. The quality of the curve fitting was measured using root-mean-square error (rmse) goodness of fit measurement, and data with fits with an rmse >15 were excluded from analysis. Using this method, curve coefficients provide values for circadian period length, amplitude and phase. Wavelet analysis was performed as described^22^ using the jlab package for MATLAB^71^ and custom MATLAB scripts based on those provided by Tanya Leise at http://www.cs.amherst.edu/tleise/CircadianWaveletAnalysis.html. Luminescence time series data from all wells in a given treatment condition were averaged, detrended and denoised with a discrete wavelet transform, and analyzed for amplitude, period, and phase using an analytic (continuous) wavelet transform. In addition to circadian amplitude, period length and phase, we also calculated a parameter that we term Net Δ Phase, defined as the sum of the differences between phase values of consecutive ZT24 (9 a.m.) time-points. We used this parameter to identify false positive period increasers. For example, for psora 4 (Fig. 5F), there is a phase advance for only the first circadian oscillation. The wavelet analysis annotates this drug as a period increaser because the time between the first two peaks is greater than 24 hours, but the Net Δ Phase value associated with this sample indicates that the period increase value is a false positive. This value is somewhat noisy, and was only used to identify false positive period increasers.

### Reverse transcription-quantitative PCR

Larval zebrafish were raised on a 14:10 hour LD cycle at 22 °C with lights on from 9 a.m. to 11 p.m. until 3 p.m. at 6 days post fertilization, at which point they were shifted into DD. Total RNA was then isolated using RNeasy (74106, Qiagen) from 24 pooled larvae every 6 hours at the indicated times. cDNA was synthesized from 5 μg total RNA using Superscript III Reverse Transcriptase (18080-051, Invitrogen) and quantitative PCR was carried out using SYBR green master mix (4364346, Life Technologies) in triplicate on an ABI PRISM 7900HF (Life Technologies) instrument. ΔCt was calculated using *actin* as a reference gene. Relative expression levels were calculated using the 2^−ΔΔCt^ method by normalizing to the sample with the highest ΔCt value for each gene^72^. Primers for amplification: *actin*, 5′-TCCTCCCTGGAGAAGAGCTATG-3′ and 5′-TCCATACCCAGGAAGGAAGG-3′. *per3*, 5′-CTCCAGCTTTCACAGCACTCA-3′ and 5′-ACGCTTCTTCATCTCCTGCAC-3′. *per1b*, 5′-ATCCAGACCCCAATACAAC-3′ and 5′-GGGAGACTCTGCTCCTTCT-3′.

## Supplementary information

Supplementary Information

## Data Availability

The datasets generated and analyzed in the current study are available from the corresponding author on reasonable request.
