# Supplementary material for: Identification of pathways that regulate circadian rhythms using a larval zebrafish small molecule screen
Source: Sci Rep. 2019 Aug 27;9:12405. doi: 10.1038/s41598-019-48914-7 (PMC6712016; doi:10.1038/s41598-019-48914-7)
Supplement: Supplementary file 1 — Supplementary Information [file 41598_2019_48914_MOESM1_ESM.pdf]

Identification of pathways that regulate circadian rhythms using a larval zebrafish small molecule screen

Eric A. Mosser[1,†], Cindy N. Chiu[1,#], T. Katherine Tamai[2], Tsuyoshi Hirota[3,4], Suna Li[1], May Hui[1], Amy Wang[1], Chanpreet Singh[1], Andrew Giovanni[5,^], Steve A. Kay[6] and David A. Prober[1,*]

[1]Division of Biology and Biological Engineering, California Institute of Technology, Pasadena, CA, 91125; [2]Centre for Cell and Molecular Dynamics, Department of Cell and Developmental Biology, University College London, London, United Kingdom, WC1E 6BT; [3]PRESTO, JST, Nagoya, Japan, 464-8601; [4]Institute of Transformative Bio-Molecules, Nagoya University, Nagoya, Japan, 464-8601; [5]Sunovion Pharmaceuticals, Marlborough, MA, 01752; [6]Department of Neurology, Keck School of Medicine, University of Southern California, Los Angeles, CA, 90089.

† Program in Biological Sciences, Northwestern University

Department of Neurobiology, Northwestern University

^ Deceased

*** Corresponding author:** David A. Prober (email: dprober@caltech.edu)

Figure S1

Figure S1. Clustergram summarizing results of the drug screen. All drugs that affected at least one of the measured circadian parameters by at least 3 standard deviations from same-plate DMSO controls were analyzed using hierarchical clustering and are depicted in this figure. The same clustergram is shown without drug annotations in **Fig. 1D**.

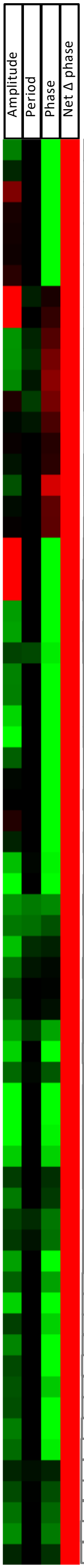

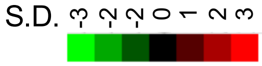

Thiophene	prostaglandin agonist
BX795	PDK1 inhibitor
Nisodipine	calcium channel blocker
GW-7647	PPARalpha activator
PI3K gamma inhibitor	PI3K gamma inhibitor
Haloperidol	D2 receptor antagonist
AS 252424	PI3K inhibitor
Felodipine	L-type Ca channel inhibitor
Mefenamic Acid	NSAID
Terazosin	alpha adrenergic antagonist
Etomidate	GABA-A activator
Ivermectin	nicotinic ACh receptor modulator
Penitrem A	potassium channel inhibitor
GC25	
PCA4248	PAF antagonist
Ethinyl estradiol	estrogen
GW-7647	PPARalpha activator
147 24 0	H1R antagonist
Fluvoxamine	SSRI
UK418	
Clomipramine	SSRI
Nisoldipine	Ca channel blocker
Tyrphostin 8	calcineurin inhibitor
Vinpocetin	PDE inhibitor
Tranylcypromine	MAOI
Suloctidil	platelet aggregation inhibitor
CGP 13501	GABA-B activator
L 703606	NK1 receptor antagonist
Dofetilide	potassium channel blocker
GW-7647	PPARalpha activator
Pimozide	D2 receptor antagonist
Tolcapone	COMT inhibitor
Prasterone	glucocorticoid
Acetyl farnesyl cysteine	farnesylation inhibitor
Aprindine HCl	hERG channel blocker
Efavirenz	reverse transcriptase inhibitor
CyPPA	potassium channel activator
Chlorophyllide	antineoplastic
JFD00244	SIRT inhibitor
Ivermectin	nicotinic ACh receptor modulator
Clinafoxacin	antibiotic
Oxybutynin Chloride	mAch antagonist
Hydroxycampothecin	topoisomerase 1 inhibitor
Chlorpheniramine maleate	H1R antagonist
Chlorprothixene	D2 receptor antagonist
Lumiracoxib	NSAID
Sertaline	SSRI
Roxithromycin	antibiolic
Lasalocid	antibacterial
Deprenyl	MAO-B inhibitor
Daunorubicin	antineoplastic
WB 4101	alpha1A adrenoceptor inhibitor
Anethole trithione	increases cholinergic response
Candicidin	antifungal
CGP 7930	GABA-B activator
Clothiapine	dopamine receptor antagonist
Nocodazole	tubulin polymerization inhibitor
Atomoxetine	SNRI
Vindesine sulfate	anti-mitotic
Sertraline HCl	SSRI
Roxithromycin	antibiotic
Promazine HCl	D2 receptor antagonist
Piperidolate HCl	antispasmodic
GC248	
Pimozide	calcium channel antagonist
Prozac	SSRI
Phenyl aminosalicylate	
Deprenyl	MAO-B inhibitor

Figure S1 (continued)

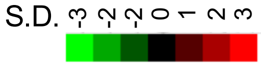

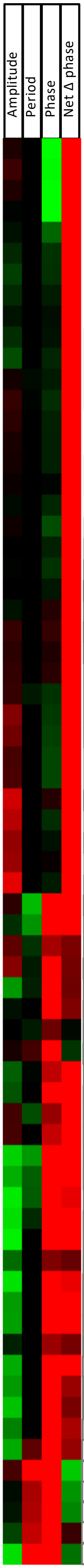

Azelastine HCl	H1R antagonist
Bepridil	calcium channel blocker
Mianserin	tetracyclic antidepressant
Danazol	testosterone
GC114	
Mebendazole	ACh esterase inhibitor
Clotrimazole	antifungal
Vinpocetine	PDE1 inhibitor
Pyrethrins	insecticide
Vorinostat	histone deacetylase inhibitor
Etazolate HCl	PDE inhibitor
Ethopropazine HCl	butyrylcholinesterase inhibitor
Niguldipine	calcium channel blocker
Trifluperidol	dopamine receptor antagonist
PAC 1	caspase 3 inhibitor
Isotharine mesylate	beta-andrenoceptor agonist
Hydroflumethiazide	antihypertensive
Pfizerpen	antibiotic
IC 261	CK1 inhibitor
Hexylresorcinol	antihelmintic
CGS 12066A	5-HT1B antagonist
Gemfibrozil	PPARalpha antagonist
Bifemelane	MAO inhibitor
UK57	H1R antagonist
Paroxetine	SSRI
Beta-estradiol	estrogen
Tolfenamic acid	prostaglandin antagonist
Haloperidol	D2 receptor antagonist
AMG 9810	TRPV1 receptor antagonist
K 185	melatonin receptor antagonist
Dioxybenzone	sunscreen
Oxaprozin	NSAID
Lyso C16 PAF	platelet activating factor
Mebendazole	ACh esterase inhibitor
Asarylaldehyde	fly attractant
Nimodipine	L-type Ca channel inhibitor
Tolterodine	antimuscarinic
Chlorpheniramine	H1R antagonist
Sorafenib	tyrosine kinase inhibitor
Canrenone	aldosterone antagonist
Chenodiol	cholesterol synthesis inhibitor
Tacrolimus	FKBP ligand
Oxaprozin	NSAID
Aminopyrine	analgesic
Flutamide	non-steroidal anti-androgen
Butaclamol	dopamine receptor antagonist
ALX 5407	glycine transporter inhibitor
Manidipine	calcium channel blocker
Verapamil	L-type Ca channel inhibitor
Paroxetine HCl	SSRI
Butaclamol	dopamine receptor antagonist
Atropine	mACh antagonist
Citalopram	SSRI
Flufenamic acid	NSAID
Clozapine	dopamine receptor antagonist
Duloxetine	SNRI
Taurine	glycine receptor agonist
Rifaximin	antibiotic
CAS 879127-07-8	EGFR inhibitor
Vincristine sulfate	microtubule assembly inhibitor
Vinblastine	microtubule assembly inhibitor
Amsacrine	topoisomerase inhibitor
Lorglumide	CCK receptor antagonist
Psora-4	Kv1.3 channel inhibitor
Gefitinib	EGFR inhibitor
Benzydamine	NSAID
Chlorphenyl dichloroethane	antiadrenal
S Nitroso N acetylpenicillamine	nitric oxide donor

Figure S1 (continued)

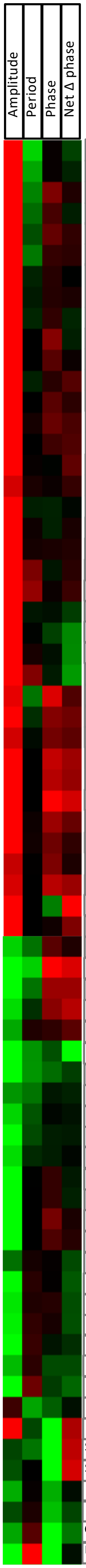

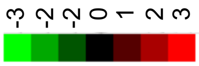

Cyclosporin	immunosuppressant
Pravadoline	NSAID
SC 51322	EP1 receptor antagonist
SU-6656	Src kinase inhibitor
Ibuprofen	NSAID
Nimodipine	L-type Ca channel inhibitor
Tivozanib	VEGFR inhibitor
Piroxicam	NSAID
Denbufylline	PDE inhibitor
SC 19220	EP1 receptor antagonist
SU-6656	Src kinase inhibitor
Chloroxine	Antibacterial
Oxaprozin	NSAID
SU-6656	Src kinase inhibitor
Meloxicam	NSAID
Rapamycin	mTOR inhibitor
WIN 62577	NK1 receptor antagonist
Rapamycin	mTOR inhibitor
Ibuprofen	NSAID
Polymyxin B sulfate	antibiotic
PF562271	FAK inhibitor
Dibucaine HCl	anesthetic
Motesanib	tyrosine kinase inhibitor
Salmeterol xinafoate	beta2 adrenergic receptor agonist
Rapamycin	mTOR inhibitor
Naproxen	NSAID
Rocuronium bromide	AChR antagonist
Ibuprofen	NSAID
Adapalene	retinoid
Estradiol acetate	estrogen
Cyproterone acetate	antiandrogen
Pravadoline	NSAID
Nalidixic acid	antibiotic
AV951	VEGFR inhibitor
VEGFR inhibitor IV	VEGFR inhibitor
Tivozanib	VEGFR inhibitor
Chloroxine	antibiotic
AC220	FLT3 inhibitor
Cdk1/2 inhibitor III	Cdk1/2 inhibitor
Orphenadrine	anticholenergic
Nonoxynol 9	spermatocide
Indirubin-3-monoxime	GSK3-beta inhibitor
Pyrantel pamoate	neuromuscular blocking agent
Pregnenolone sulfate	GABA-A antagonist
Celecoxib	NSAID
Mitomycin C	antibiotic
Resveratrol	sirtuin activator
Axitinib	tyrosine kinase inhibitor
Fenbufen	NSAID
Tranilast	PGE2 inhibitor
CP-868388	PPARalpha agonist
CP-868388	PPARalpha agonist
CP-868388	PPARalpha agonist
Nicergoline	alpha-adrenergic antagonist
5 Nitro-2-3-phenylpropyl benzoic acid	Cl channel blocker
Pranlukast	leukotriene receptor antagonist
Merbromin	antibacterial
Fendendazole	antihelmintic
Daunorubicin	antineoplastic
Pyrvinium pamoate	antihelmintic
Resveratrol	sirtuin activator
Naproxen	NSAID
Spironolactone	diuretic
Sumatriptan succinate	5HT receptor agonist
Pararosaniline	antihelmintic
Rosiglitazone	PPAR agonist
Cis Piperidine	NMDAR agonist
Progesterone	estrogen receptor agonist

Figure S1 (continued)

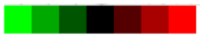

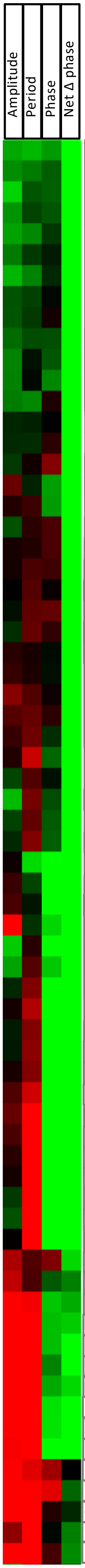

Proflavine	bacteriostatic
Chlorocresol	methylphenol
Nabumetone	NSAID
Valganciclovir	antiviral
Nevirapine	reverse transcription inhibitor
Estradiol acetate	estrogen
Fenoterol HBr	beta2-adrenoreceptor agonist
3-tropanyl-3-5-dichlorobenzoate	5-HT3 receptor antagonist
Aconitine	Na channel agonist
Budesonide	glucocorticoid
Flupenthixol	dopamine receptor antagonist
Oxolamine citrate	cough suppressant
UK22	
5-alpha-androstan	androgen
Baccatin III	precursor to paclitaxel
Lomifylline	PDE inhibitor
Cardene	Ca channel blocker
Eicosadienoic acid	lipoxygenase inhibitor
Okadaic acid	PP1 PP2A phosphatase inhibitor
Piribedil	dopamine receptor agonist
Nefazodone	5-HT2 receptor antagonist
Temefos	insecticide
Aklavine HCl	antibacterial
Amlexanox	anti-inflammatory
Sphingosine	PKC inhibitor
Urapidil HCl	5HT1A receptor agonist
Hydrocortisone	glucocorticoid
Finasteride	type II 5a reductase inhibitor
U 50488	k-opioid agonist
N-Phenylanthranilic	misc. channels
R Desmethyldeprenyl	MAO-B inhibitor
Selamectin	antiparasitic
Beclomethasone	arachidonic acid inhibitor
Azaperone	dopamine antagonist
UK420	
Chlormadinone	steroidal progestin
Resveratrol	sirtuin activator
Metformin	AMPK activator
Resveratrol	sirtuin activator
17 Allylamino geldanamycin	HSP-90 inhibitor
Resveratrol	sirtuin activator
Atropine methyl Br	mACh receptor antagonist
SNS 032	CDK inhibitor
PP2	Src kinase inhibitor
hydrocortisone	glucocorticoid
Halometasone	corticosteroid
Isonicotinamide	sirtuin activator
Caffeic acid phenethyl ester	NFkB inhibitor
Resveratrol	sirtuin activator
Resveratrol	sirtuin activator
Betamethasone	corticosteroid
Molsidomine	vasodilator
Isradipine	L-type Ca channel inhibitor
Melengestrol	steroidal progestin
Warfarin	vitamin K reductase inhibitor
Cortisone acetate	corticosteroid
Prednisolone	MAPK inhibitor
Dicumarol	anticoagulant
Oleic acid	PKC activator
Dexamethasone	glucocorticoid
Metformin	AMPK activator
AICAR	AMPK activator
Corticosterone	corticosteroid
TMB HCl	calcium antagonist
SB415286	GSK3 inhibitor
Adapalene	retinoid
AICAR	AMPK activator
SU-6656	Src kinase inhibitor

Figure S1 (continued)

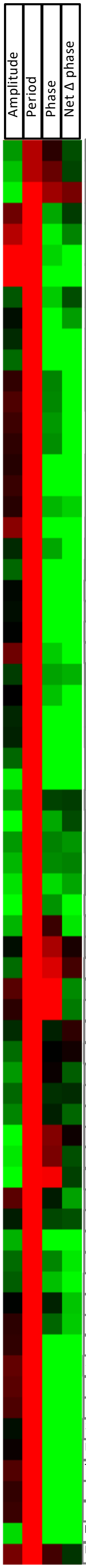

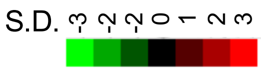

Capsaicin	TRPV1 activator
BX795	PDK1 inhibitor
Clofibrate	lipoprotein lipase activator
PIM1/2 kinase inhibitor IV	PIM1/2 kinase inhibitor
50 22 6	glucocorticoid
Metformin	AMPK activator
Dexamethasone	glucocorticoid
SB 239063	p38 MAPK inhibitor
Roscovitine	CDK inhibitor
UK343	glucocorticoid
Goat's rue extract	
Proglumide	CCK receptor antagonist
Propafenone	K channel blocker
Donepezil	AChE inhibitor
Tetrabenazine	VMAT inhibitor
Resveratrol	sirtuin activator
CGP 74514A	CDK1 inhibitor
Resveratrol	sirtuin activator
TG003	Cdc2-like kinase inhibitor
Physostigmine	AChE inhibitor
Triamcinolone	glucocorticoid
UK326	glucocorticoid
Isonicotinamide	sirtuin activator
Acetylsalicylic acid	NSAID
TOVOK	HER2 and EGFR antagonist
H 89	PKA inhibitor
Natriben methanesulfonate	delta2 opiod receptor antagonist
Resveratrol	sirtuin activator
Resveratrol	sirtuin activator
Rhodblock 6	Rho kinase inhibitor
GNE 490	PI3K inhibitor
Oxibendazole	antihelmintic
Zafirlukast	leukotriene receptor antagonist
Arecaidine	mACh receptor agonist
Phenindione	anticoagulant
Amifostine	radioprotective agent
Phloretin	L-type Ca channel blocker
AS 252424	PI3K gamma inhibitor
Ro 31 8220	PKC inhibitor
Aztreonam	cell wall synthesis inhibitor
Bosutinib	tyrosine kinase inihbitor
KU0063794	mTORC1/2 inhibitor
PF04217903	C-Met inihibitor
SCH 51344	Ras/Rac transformation blocker
Yohimbine	alpha-2 adrenoreceptor inhibitor
Raloxifene	estrogen receptor modulator
AG 9	EGFR kinase inhibitor
Climbazole	antifungal
Cerulenin	fatty acid biosynthesis inhibitor
Indirubin 3 monoxime	GSK-3b inhibitor
Doxazosin	alpha-1 adrenoceptor
Enalaprilat	ACE inhibitor
Deflazacort	glucocorticoid
Ranitidine	H2R antagonist
RU 24969	5HTA1/A2 receptor agonist
Nortriptyline	tricyclic antidepressant
Methoctramine	M2 mACh receptor antagonist
Metformin	AMPK activator
SSR112050	CK1 inhibitor
SSR112050	CK1 inhibitor
SSR112050	CK1 inhibitor
SSR112050	CK1 inhibitor
N9 Isopropylolomoucine	CDC-2 kinase inhibitor
Tracazolate	non-benzodiazepine
Roscovitine	CDK inhibitor
TAK 715	P38 MAPK inhibitor
Roscovitine	CDK inhibitor
Dehydroepiandrosterone	corticosteroid

Figure S1 (continued)

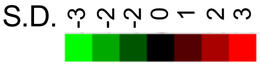

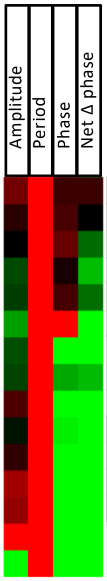

ABT 737	Bcl-2 inhibitor
VEGF receptor TK inhibitor II	VEGFR inhibitor
MEK1/2 inhibitor II	MEK1/2 inhibitor
GW-7647	PPARalpha activator
Oxatomide	H1R antagonist
BI 2536 R	PLK inhibitor
BX912	PDK1 inhibitor
Gefitinib	EGFR inhibitor
Bohemine	CDK1 inhibitor
Vandetanib	EGFR inhibitor
Isonicotinamide	sirtuin activator
Isonicotinamide	sirtuin activator
Isonicotinamide	sirtuin activator
Isonicotinamide	sirtuin activator
Chlorzoxazone	NO synthetase inhibitor

Figure S2

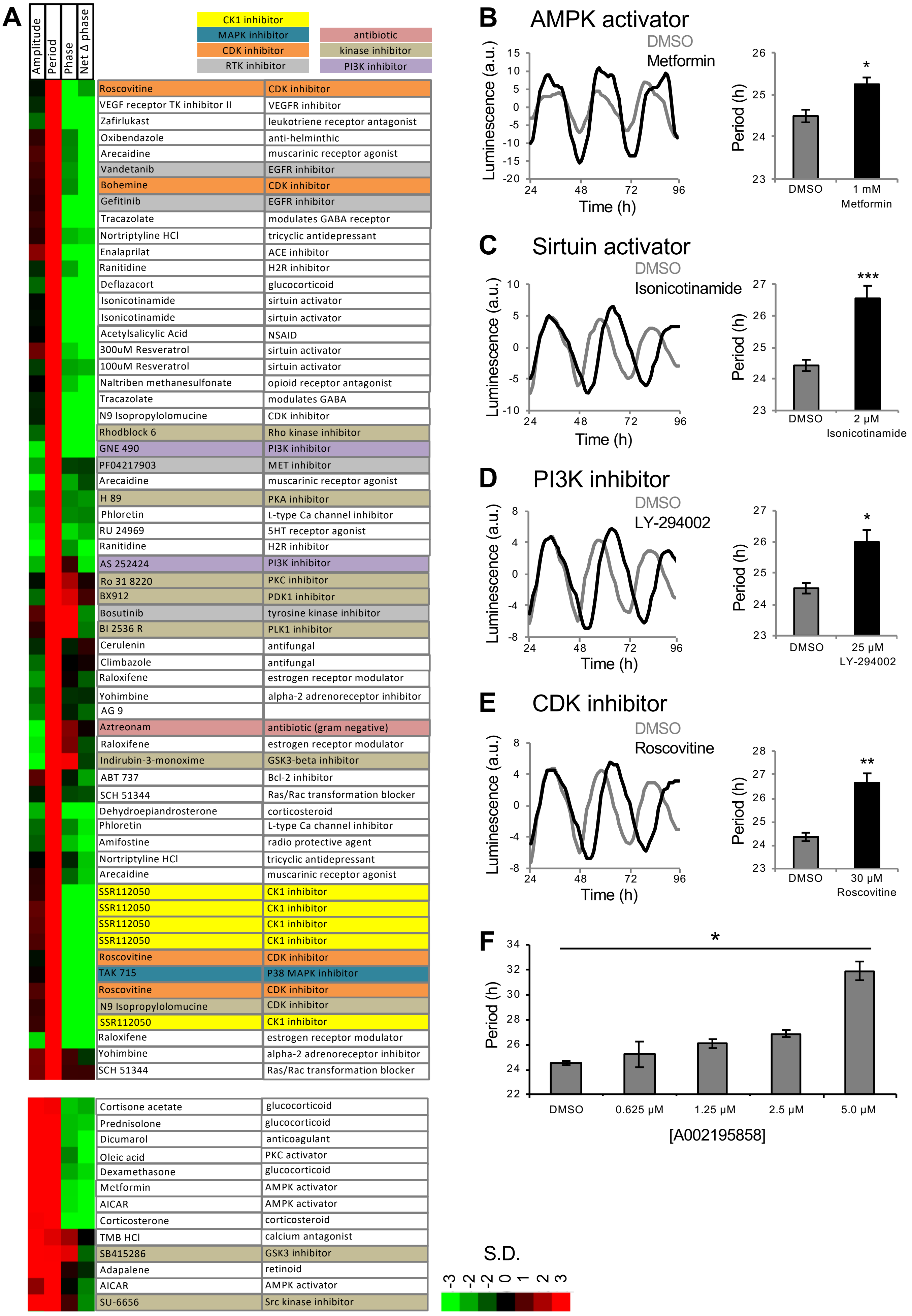
A
Amplitude
Period
Phase
Net Δ phase
CK1 inhibitor
MAPK inhibitor
CDK inhibitor
RTK inhibitor
antibiotic
kinase inhibitor
PI3K inhibitor
Roscovitine | CDK inhibitor
VEGF receptor TK inhibitor II | VEGFR inhibitor
Zafirlukast | leukotriene receptor antagonist
Oxibendazole | anti-helminthic
Arecaidine | muscarinic receptor agonist
Vandetanib | EGFR inhibitor
Bohemine | CDK inhibitor
Gefitinib | EGFR inhibitor
Tracazolate | modulates GABA receptor
Nortriptyline HCl | tricyclic antidepressant
Enalaprilat | ACE inhibitor
Ranitidine | H2R inhibitor
Deflazacort | glucocorticoid
Isonicotinamide | sirtuin activator
Isonicotinamide | sirtuin activator
Acetylsalicylic Acid | NSAID
300uM Resveratrol | sirtuin activator
100uM Resveratrol | sirtuin activator
Naltriben methanesulfonate | opioid receptor antagonist
Tracazolate | modulates GABA
N9 Isopropylolomucine | CDK inhibitor
Rhodblock 6 | Rho kinase inhibitor
GNE 490 | PI3K inhibitor
PF04217903 | MET inhibitor
Arecaidine | muscarinic receptor agonist
H 89 | PKA inhibitor
Phloretin | L-type Ca channel inhibitor
RU 24969 | 5HT receptor agonist
Ranitidine | H2R inhibitor
AS 252424 | PI3K inhibitor
Ro 31 8220 | PKC inhibitor
BX912 | PDK1 inhibitor
Bosutinib | tyrosine kinase inhibitor
BI 2536 R | PLK1 inhibitor
Cerulenin | antifungal
Climbazole | antifungal
Raloxifene | estrogen receptor modulator
Yohimbine | alpha-2 adrenoreceptor inhibitor
AG 9
Aztreonam | antibiotic (gram negative)
Raloxifene | estrogen receptor modulator
Indirubin-3-monoxime | GSK3-beta inhibitor
ABT 737 | Bcl-2 inhibitor
SCH 51344 | Ras/Rac transformation blocker
Dehydroepiandrosterone | corticosteroid
Phloretin | L-type Ca channel inhibitor
Amifostine | radio protective agent
Nortriptyline HCl | tricyclic antidepressant
Arecaidine | muscarinic receptor agonist
SSR112050 | CK1 inhibitor
SSR112050 | CK1 inhibitor
SSR112050 | CK1 inhibitor
SSR112050 | CK1 inhibitor
Roscovitine | CDK inhibitor
TAK 715 | P38 MAPK inhibitor
Roscovitine | CDK inhibitor
N9 Isopropylolomucine | CDK inhibitor
SSR112050 | CK1 inhibitor
Raloxifene | estrogen receptor modulator
Yohimbine | alpha-2 adrenoreceptor inhibitor
SCH 51344 | Ras/Rac transformation blocker
Cortisone acetate | glucocorticoid
Prednisolone | glucocorticoid
Dicumarol | anticoagulant
Oleic acid | PKC activator
Dexamethasone | glucocorticoid
Metformin | AMPK activator
AICAR | AMPK activator
Corticosterone | corticosteroid
TMB HCl | calcium antagonist
SB415286 | GSK3 inhibitor
Adapalene | retinoid
AICAR | AMPK activator
SU-6656 | Src kinase inhibitor
S.D.
-3 -2 -2 0 1 2 3
B AMPK activator
DMSO
Metformin
Luminescence (a.u.)
Time (h)
Period (h)
DMSO
1 mM Metformin
*
C Sirtuin activator
DMSO
Isonicotinamide
Luminescence (a.u.)
Time (h)
Period (h)
DMSO
2 μM Isonicotinamide
***
D PI3K inhibitor
DMSO
LY-294002
Luminescence (a.u.)
Time (h)
Period (h)
DMSO
25 μM LY-294002
*
E CDK inhibitor
DMSO
Roscovitine
Luminescence (a.u.)
Time (h)
Period (h)
DMSO
30 μM Roscovitine
**
F
Period (h)
*
DMSO
0.625 μM
1.25 μM
2.5 μM
5.0 μM
[A002195858]

Figure S2. Drugs that increased *Tg(per3:luc)* luminescence circadian period length. (**A**) Clusters of drugs that increased period length by at least 3 standard deviations from the mean of same-plate DMSO controls is shown. Color-coding indicates drug classes. Compounds that increased period length include AMPK activator metformin (**B**), sirtuin activator isonicotinamide (**C**), PI3K inhibitor LY-204002 (**D**) and CDK inhibitor roscovitine (**E**). Line graphs show mean and bar graphs show mean ± SEM for 8 animals. **P*<0.05, ***P*<0.01, ****P*<0.001 by ANOVA with Tukey's test. (**F**) Treatment of Rat-1 fibroblasts stably transfected with a *mPer1-luc* reporter construct with the CK1 inhibitor A002195858 resulted in significantly increased period length at 5 μM. Mean ± SEM for triplicate samples are shown. **P*<0.05 by Kruskal-Wallis test corrected for multiple comparisons to DMSO using the Dunn test.

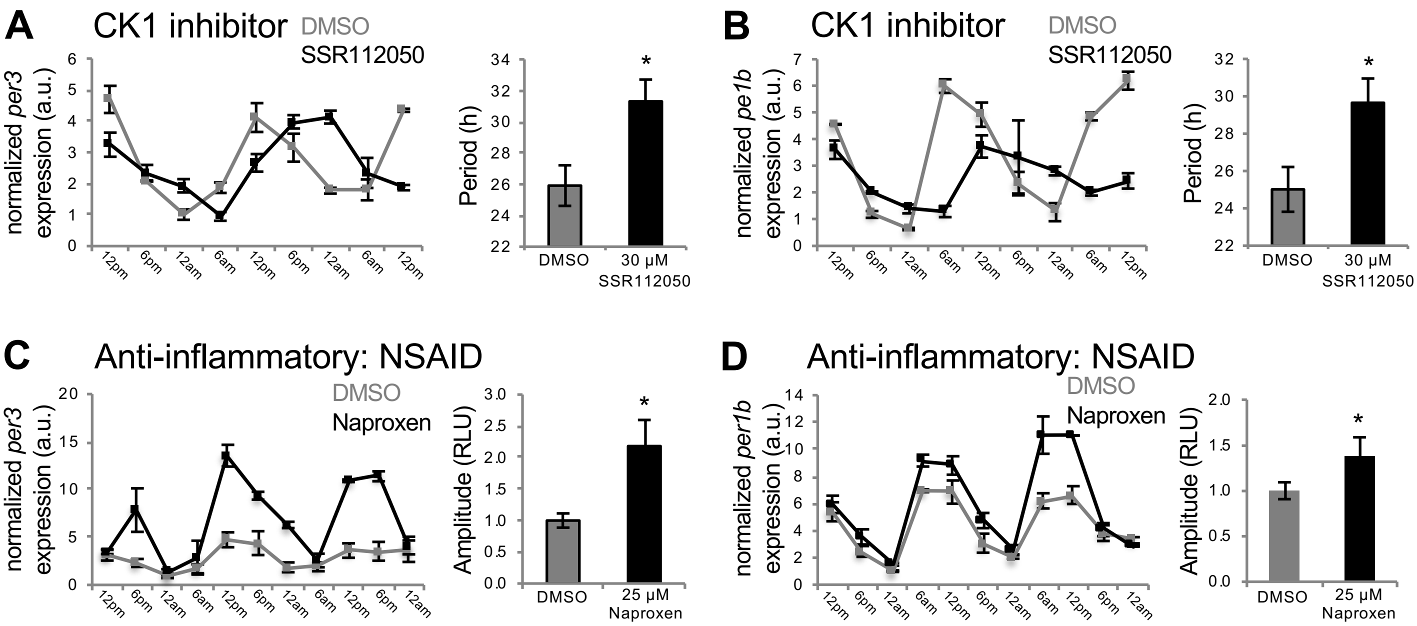

Figure S3. Confirmation of drug-induced luminescence phenotypes using RT-qPCR. CK1 inhibitor SSR112050 increased period length of *per3* (**A**) and *per1b* (**B**) circadian oscillations. Anti-inflammatory naproxen increased amplitude of *per3* (**C**) and *per1b* (**D**) circadian oscillations. Mean ± SEM is shown. **P*<0.05 by ANOVA with Tukey's test.

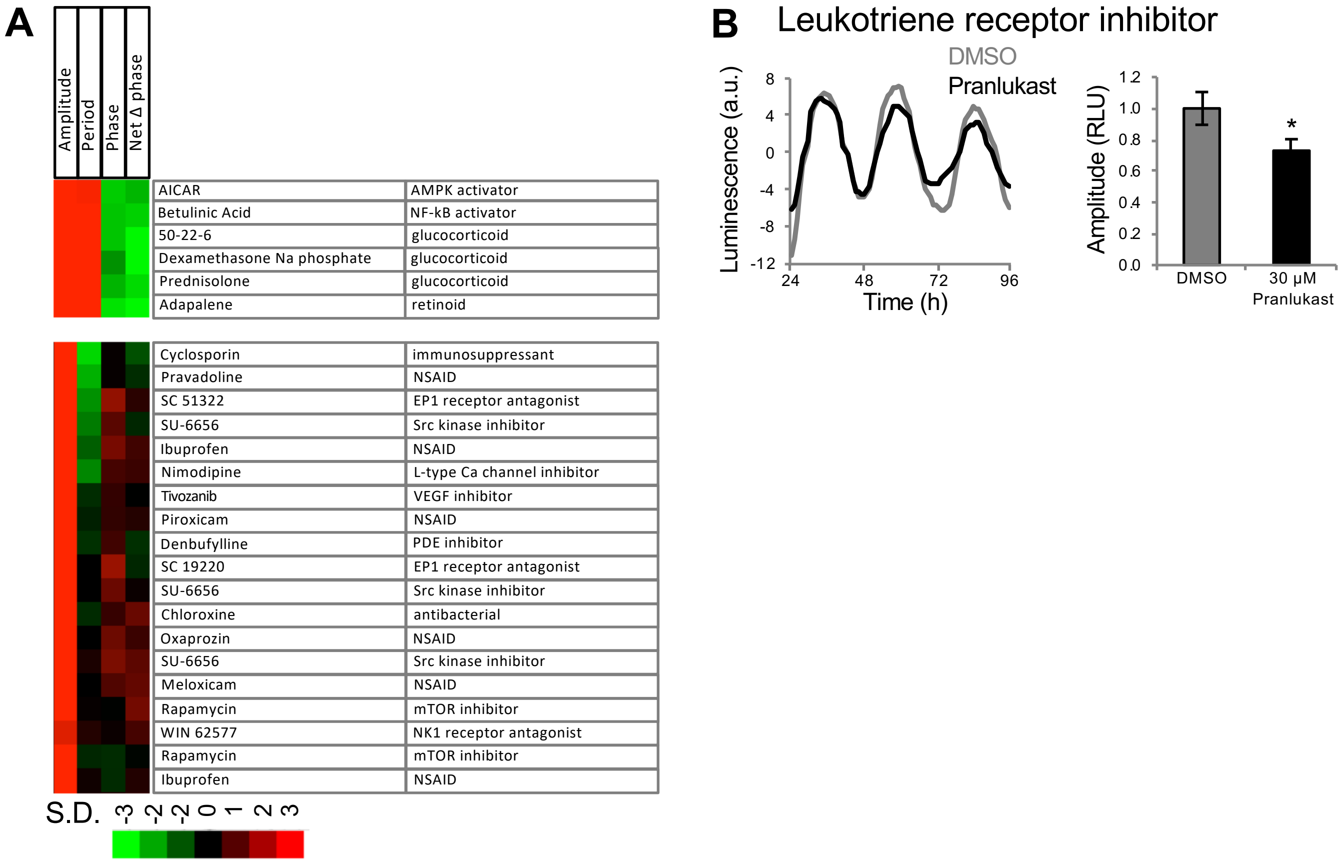

Figure S4. Drugs that affected *Tg(per3:luc)* luminescence circadian amplitude. (**A**) Clusters of drugs that affected circadian amplitude by at least 3 standard deviations from the mean of same-plate DMSO controls is shown. (**B**) Leukotriene receptor inhibitor pranlukast decreased circadian amplitude. **P*<0.05 by ANOVA with Tukey's test.

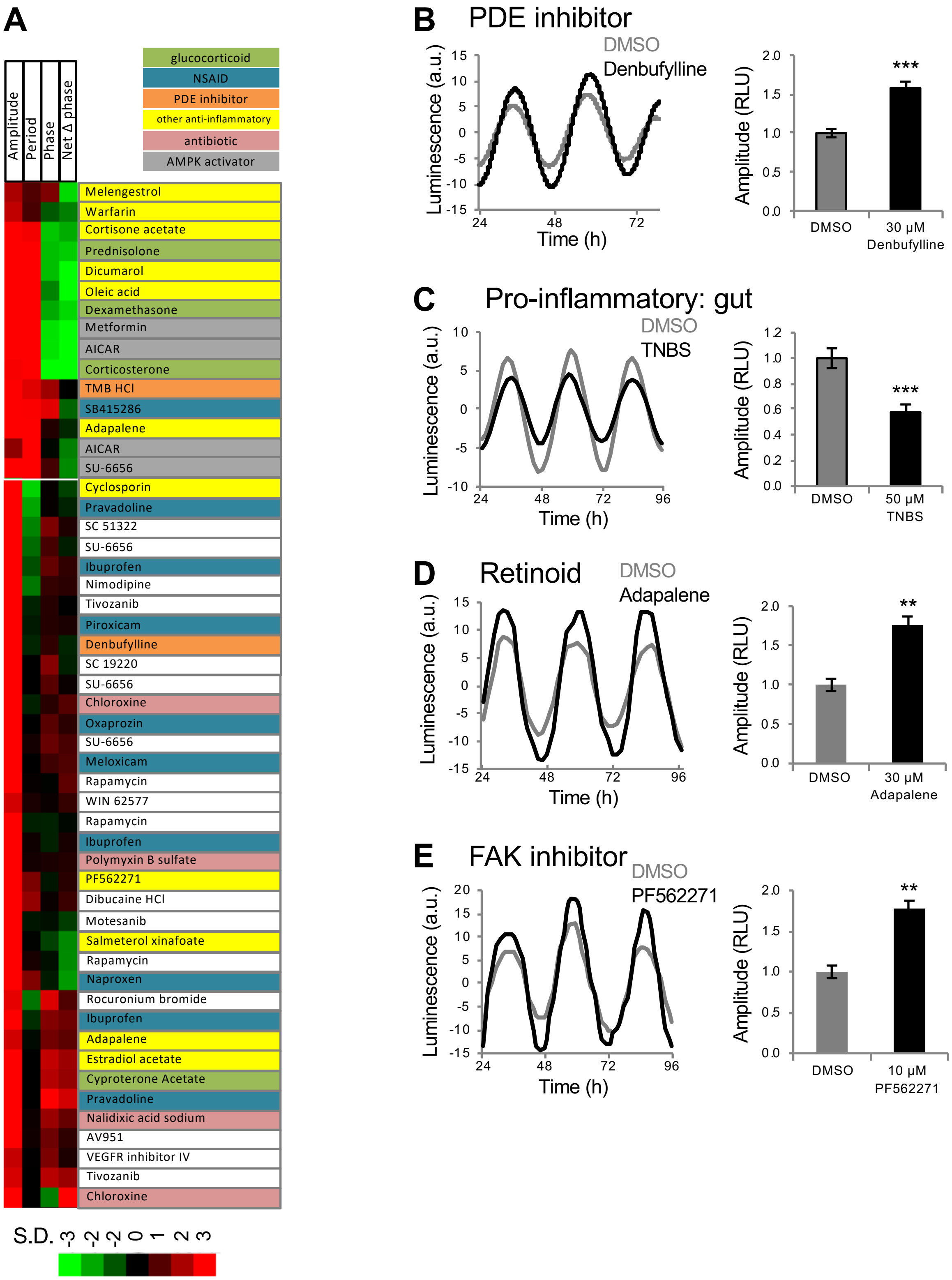

Figure S5. Inflammatory drugs that affected *Tg(per3:luc)* luminescence circadian amplitude. (**A**) A cluster of drugs that affected circadian amplitude by at least 3 standard deviations from the mean of same-plate DMSO controls is shown. Color-coding indicates drug classes. Anti-inflammatory compounds that increased circadian amplitude include PDE inhibitor denbufylline (**B**), retinoid adapalene (**D**) and FAK inhibitor PF562271 (**E**). TNBS, which induces inflammation in the gut, decreased circadian amplitude (**C**). $^{**}P<0.01$, $^{***}P<0.001$ by ANOVA with Tukey's test.

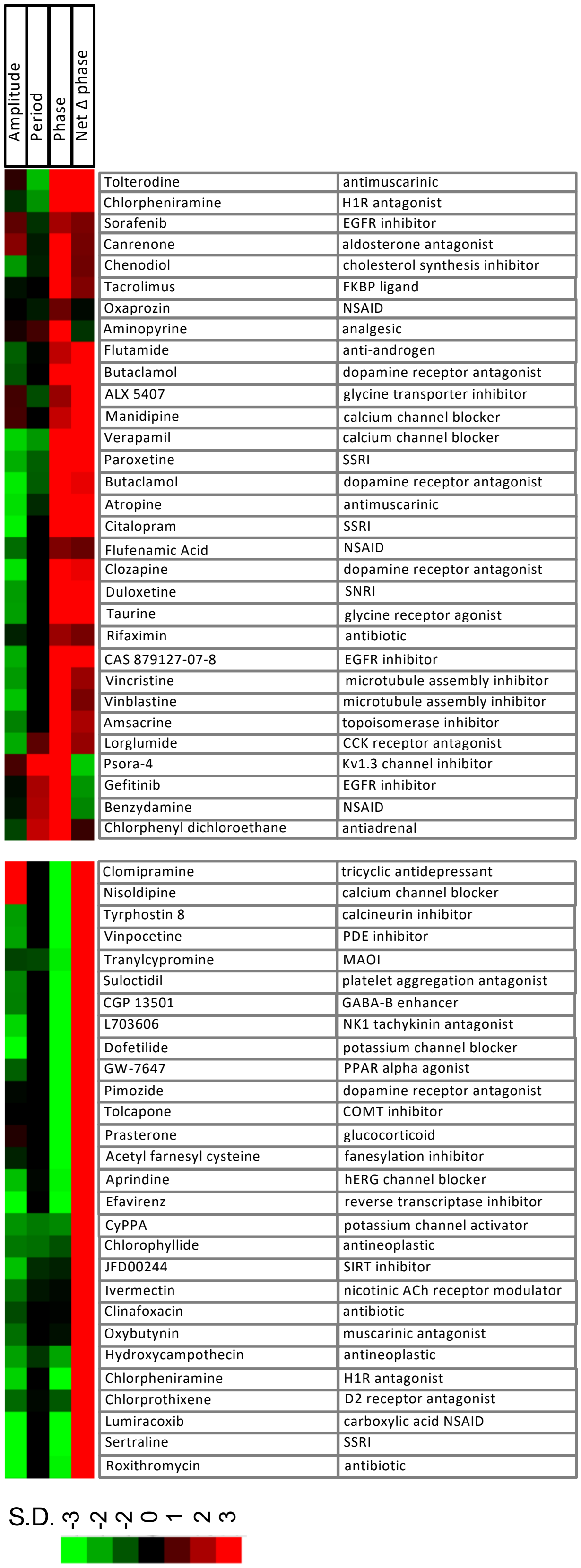

Figure S6. Clusters of drugs that affected *Tg(per3:luc)* luminescence circadian phase. Drugs that affected circadian phase by at least 3 standard deviations from the mean of same-plate DMSO controls are shown.

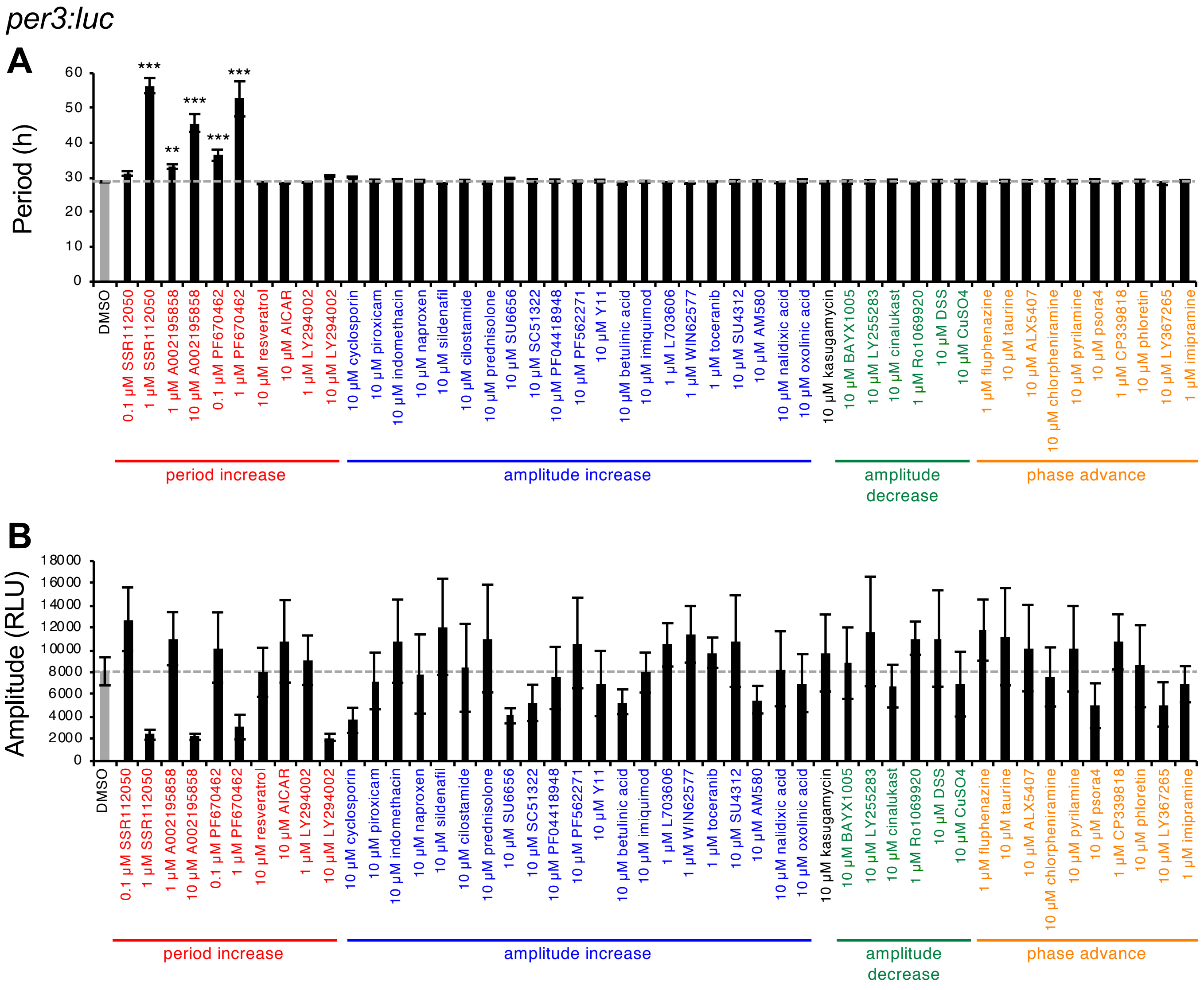

Figure S7. Effects of drugs on luminescence circadian period length and amplitude in cells derived from *Tg(per3:luc)* embryos. (**A**) Some drugs that increased period length in *Tg(per3:luc)* larvae, including SSR112050, A002195858 and PF670462, induced similar phenotypes in *per3:luc* cells, while others, such as resveratrol, AICAR and LY294002, did not. (**B**) Drugs that affected amplitude in *Tg(per3:luc)* larvae did not induce similar phenotypes in *per3:luc* cells. Each case of severely reduced luminescence amplitude was associated with drug-induced toxicity. Mean ± SEM is shown. ***P*<0.01, ****P*<0.001 for pairwise comparisons between each drug and DMSO control by Dunnett's test. Labels are colored according to drug effects in *Tg(per3:luc)* larvae.

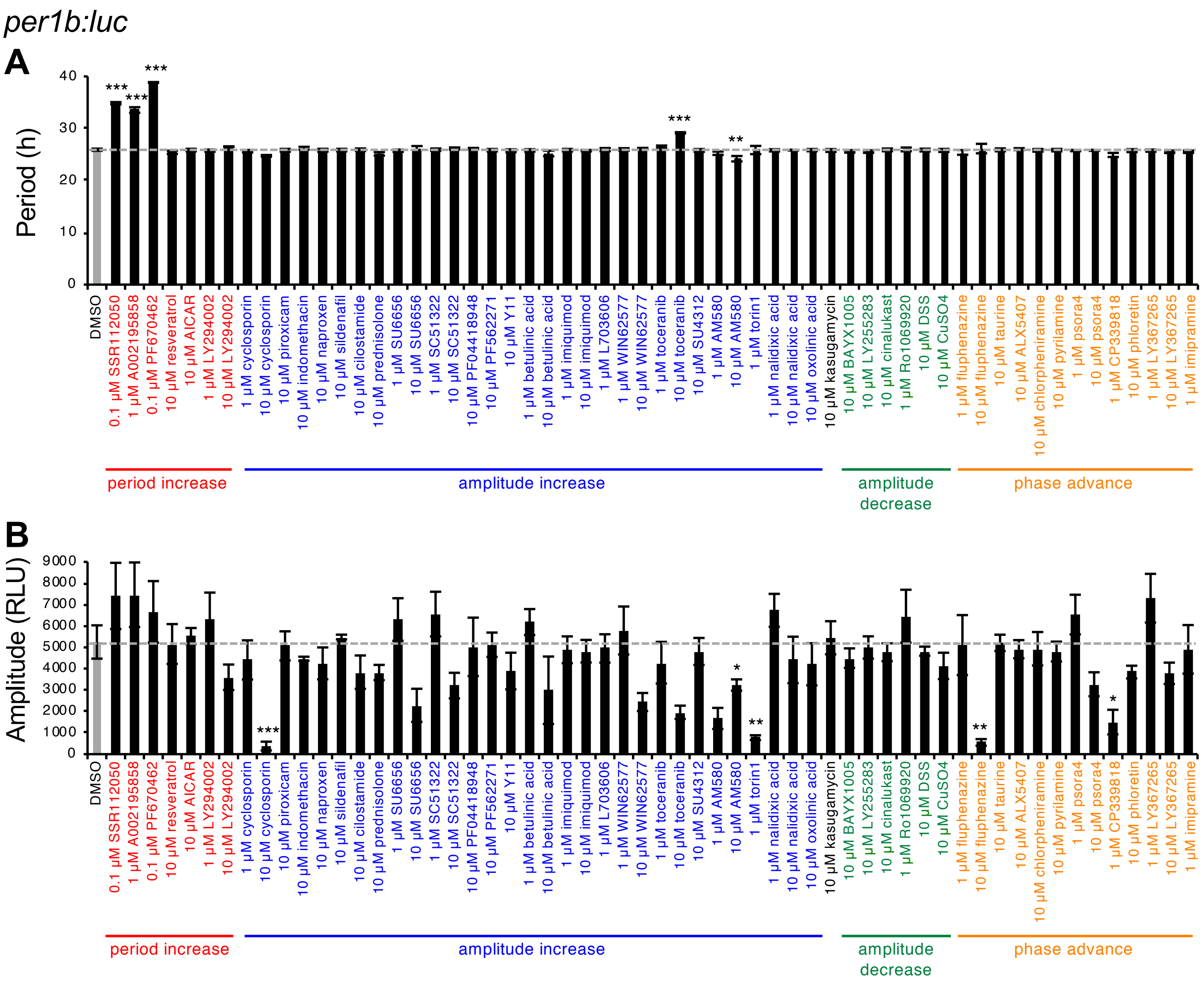

Figure S8. Effects of drugs on luminescence circadian period length and amplitude in *per1b:luc* zebrafish cells. (**A**) Some drugs that increased period length in *Tg(per3:luc)* larvae, including SSR112050, A002195858 and PF670462, induced similar phenotypes in *per1b:luc* cells, while others, such as resveratrol, AICAR and LY294002, did not. (**B**) Drugs that affected amplitude in *Tg(per3:luc)* larvae did not induce similar phenotypes in *per1:luc* cells. Each case of severely reduced circadian amplitude was associated with drug-induced toxicity. Mean ± SEM is shown. *P<0.05, **P<0.01, ***P<0.001 for pairwise comparisons between each drug and DMSO control by Dunnett's test. Labels are colored according to drug effects in *Tg(per3:luc)* larvae.

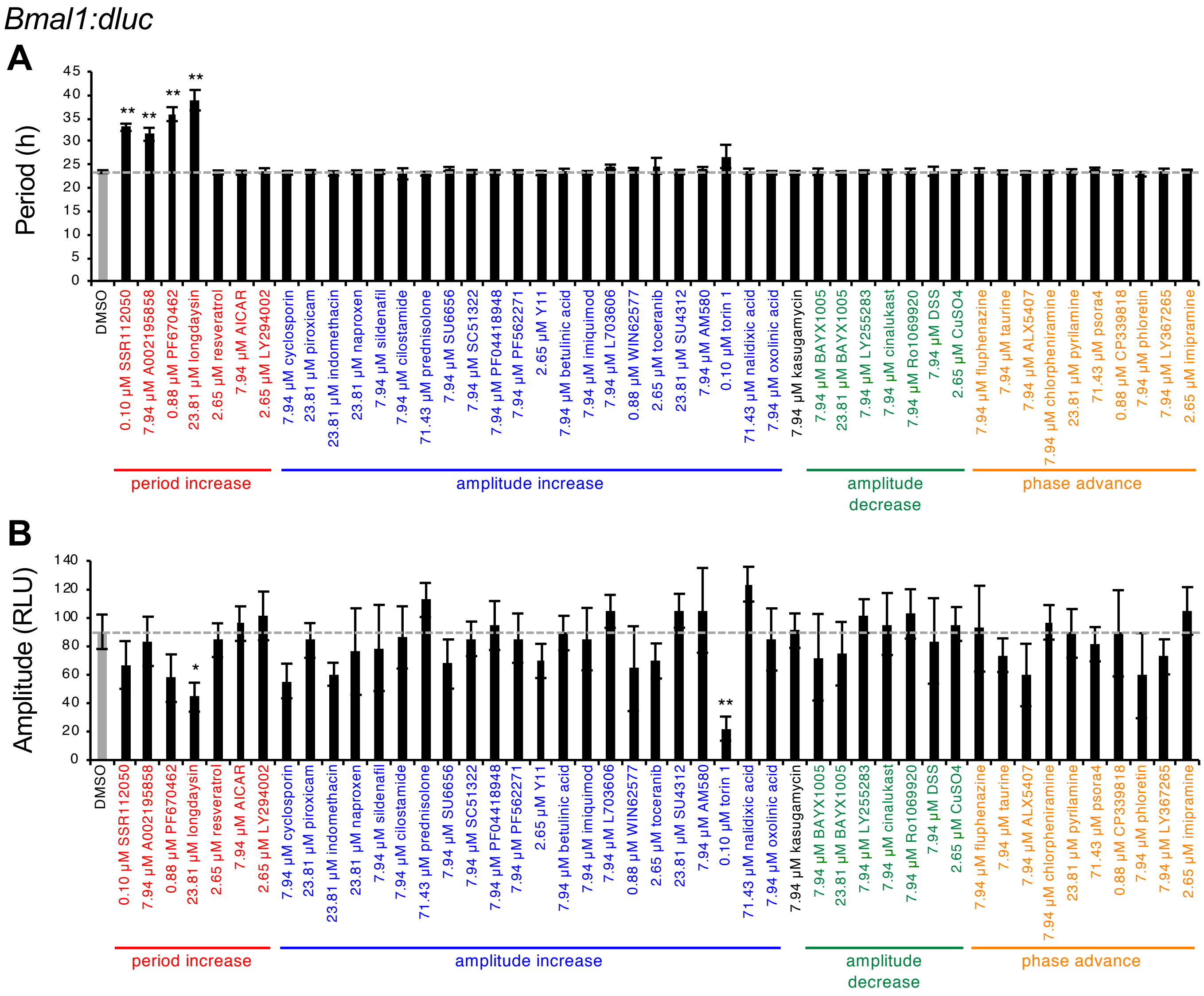

Figure S9. Effects of drugs on luminescence circadian period length and amplitude in *Bmal1:dluc* human U2OS cells. (**A**) Some drugs that increased period length in *Tg(per3:luc)* larvae, including SSR112050, A002195858 and PF670462, induced similar phenotypes in *Bmal1:dluc* cells, while others, such as resveratrol, AICAR and LY294002, did not. Longdaysin, a CK1 and ERK2 antagonist (Ref 5), was used as a positive control. (**B**) Drugs that affected amplitude in *Tg(per3:luc)* larvae did not induce similar phenotypes in *Bmal1:dluc* cells. Each case of severely reduced circadian amplitude was associated with drug-induced toxicity. Mean ± SEM is shown. *P<0.05, **P<0.01 for pairwise comparisons between each drug and DMSO control by Dunnett's test. Labels are colored according to drug effects in *Tg(per3:luc)* larvae.
